# Pollinator divergence and pollination isolation between hybrids with different floral color and morphology in two sympatric *Penstemon* species

**DOI:** 10.1038/s41598-020-64964-8

**Published:** 2020-05-15

**Authors:** Juliana Cardona, Carlos Lara, Juan Francisco Ornelas

**Affiliations:** 1 Centro de Investigación en Ciencias Biológicas, Universidad Autónoma de Tlaxcala, San Felipe Ixtacuixtla, 90120 Tlaxcala, Mexico; 2 Departamento de Biología Evolutiva, Instituto de Ecología, A.C. (INECOL), Carretera antigua a Coatepec 351, El Haya, Xalapa 91073 Veracruz, Mexico

**Keywords:** Behavioural ecology, Evolutionary ecology

## Abstract

Differential visitation of pollinators due to divergent floral traits can lead to reproductive isolation via assortative pollen flow, which may ultimately be a driving force in plant speciation, particularly in areas of overlap. We evaluate the effects of pollinator behavioral responses to variation of intraspecific floral color and nectar rewards, on reproductive isolation between two hybrid flower color morphs (fuchsia and blue) and their parental species *Penstemon roseus* and *P. gentianoides* with a mixed-pollination system. We show that pollinators (bumblebees and hummingbirds) exhibit different behavioral responses to fuchsia and blue morphs, which could result from differential attraction or deterrence. In addition to differences in color (spectral reflectance), we found that plants with fuchsia flowers produced more and larger flowers, produced more nectar and were more visited by pollinators than those with blue flowers. These differences influenced the foraging behavior and effectiveness as pollinators of both bumblebees and hummingbirds, which contributed to reproductive isolation between the two hybrid flower color morphs and parental species. This study demonstrates how differentiation of pollination traits promotes the formation of hybrid zones leading to pollinator shifts and reproductive isolation. While phenotypic traits of fuchsia and red flowers might encourage more efficient hummingbird pollination in a mixed-pollination system, the costs of bumblebee pollination on plant reproduction could be the drivers for the repeated shifts from bumblebee- to hummingbird-mediated pollination.

## Introduction

Transitions from one to another pollinator type have played a fundamental role in divergence of flower phenotypes, development of reproductive isolation among incipient species and, ultimately, in the origin and diversification of Angiosperms^1–3^. The most compelling evidence for the role of pollinators in speciation via pollinator transitions comes from studies of closely related, insect- or hummingbird-pollinated plant species^4–7^, focusing on phenotypic traits (e.g., color, morphology, aroma, floral phenology, incompatibility) that enable plants to exploit the morphology, sensory mechanisms, and nutritional needs of different types of pollinators. The floral preferences of different pollinating agents result in modifications of a suite of floral characteristics (pollinator-mediated divergent selection), which potentially lead to differentiation of the floral phenotype and produce reproductive isolation in sympatry^8–12^. Thus, plant groups that exhibit pollinator shifts are an ideal system as to understand the role of these transitions in plant species diversification and phenotypic changes of flowers over evolutionary time^13–16^.

Sister species that display floral trait differences linked to the bumblebee or the hummingbird pollination syndrome often occur in several North American plant genera^3,16–18^. These pollinator-syndrome shifts are associated with the contrasting visual color system and visual capabilities of bees (or bumblebees) and hummingbirds^19–22^, trichromatic and tetrachromatic color vision, respectively^23–25^. Bees’ photoreceptor sensitivities peak at about 350, 440 and 540 nm^23–27^. Although the shift to shorter wavelengths (reflectance spectra near 400 nm or 500 nm) allows bees to see UV radiation, long wavelength “red” radiation is poorly discriminated by bee photoreceptors^26,28^. Birds are also sensitive to UV, with some variability of their spectral sensitivity for discrimination of blue, green and red wavelengths^24,25,29^. Because of the violet sensitive (VS) color vision of hummingbirds^19^, flowers adapted to pollination by these animals should be clustered close to 600 nm^26^.

The efficiency at recognizing flowers with red and blue signals also relates to differences between hummingbirds and bumblebees in their preferences and foraging efficiencies, respectively^30^. However, if reproductive isolation is shown to be incomplete between closely related plant species, hybrid zones can form because interspecific pollen transfer prevails^31,32^. Yet, competition for pollinators can create the conditions for reproductive (prezygotic) isolation between populations at the hybrid zones^32^. The resulting selection would facilitate divergence of floral traits that would attract the most efficient pollinator type^33,34^. If two types of flower morphologies specialize on different pollinator types, hybrids are expected to have intermediate morphology (i.e., phenotypic traits that resemble both bee and hummingbird-pollination), receive few pollinator visits and to have a low pollination success because of divergent or disruptive selection on floral traits, which represent mechanisms of postzygotic reproductive isolation^31^. What remains unclear, however, are the ecological conditions driving reproductive isolation in species belonging to different clades and that are already differentiated, with mixed-pollination systems (i.e., bee and hummingbird-pollination) and forming hybrid zones when growing in sympatry.

The North American genus *Penstemon* (284 species) exhibits a great floral diversity that is mainly associated with bumblebee and hummingbird pollination^2,17,35–37^. Evolutionary transitions from insect pollination to hummingbird pollination have occurred an estimated 15–20 times during *Penstemon* diversification^5,17^. These transitions have occurred in *Penstemon* presumably because bumblebees consume pollen, depleting the number of pollen grains they transfer between flowers, and because hummingbirds are more efficient at transferring pollen (i.e., pollen carryover) than bumblebees^5,38^. Transitions to hummingbird pollination in *Penstemon* are usually associated with the evolution of bright red to magenta, long and narrow flowers that have exserted reproductive organs and produce large amounts of dilute nectar^2,17,18,35,39,40^. Yet, despite this and other changes in floral dimension and flower orientation, the majority of the pollination-syndrome transitions in *Penstemon* seem to be more easily predicted by transitions from blue-purple to red flowers^17^. These transitions involve the loss of the enzyme that is responsible for converting precursors of red pigments into those of blue pigments^41^, which strongly hinder evolutionary reversals in *Penstemon*^42^.

Transitions from blue to red flowers in *Penstemon* seem to include stages in which floral traits are intermediate between bee- and hummingbird-pollination modes and species maintain mixed-pollination systems^43–47^. The apparently intermediate floral traits in species with mixed-pollination systems might be potentially acting as mechanisms to deter bumblebee foragers^40,48–50^. If these intermediates deter bumblebee foragers, then they could be the pathways for the evolution of hummingbird-pollinated plants. Then, increased hummingbird visitation might be selected over evolutionary time, resulting in trait switches, which are important for mediating reproductive isolation, and the evolution of floral traits that may ease transitions to hummingbird pollination^7,18,37,50^. However, it is not known how these transitions occur in *Penstemon*, i.e., whether the attractiveness of intermediate floral phenotypes, expected for transitions from bee to hummingbird evolution, relate to different pollinators and the reproductive isolation among hybrids and parental species.

Here, we use two coexisting *Penstemon* hybrid flower color morphs (blue and fuchsia morphs) with mixed-pollination systems as a model system to investigate whether differential visitation of pollinators can lead to reproductive isolation. The two hybrid flower color morphs probably are the result from different pollination agencies, i.e., the fuchsia morph is the hybrid with *P*. *gentianoides* pollinated by *P*. *roseus*, while the blue hybrid has resulted from *P*. *roseus* receiving *P*. *gentianoides* pollen. Specifically, we addressed the following questions: (1) Are the parental species, *P. gentianoides* and *P. roseus*, and their hybrids distinguishable by bumblebees and hummingbirds? (2) Do parental species and hybrid flower color morphs differ in their detectability when considering the visual color abilities of bumblebees and hummingbirds? (3) Is the natural variation in floral and nectar traits associated with differences in foraging behavior and pollinator effectiveness of floral visitors? (4) Do female reproductive success and reproductive isolation (*RI*) differ between the flower color morphs and parental species?

## Results

### Floral biology

On average, flowers of the fuchsia morph were significantly larger than those of the blue morph (*P* < 0.05), except filament (stamen) length (Suppl. Table S1). Flower longevity, ranging from 6 to 14 days for all plants, differed between hybrid flower color morphs (fuchsia = 9.72 ± 0.343, blue = 10.17 ± 0.205; *F*_1, 78_ = 7.36, *P* = 0.009). Stigmatic receptivity, highest between the 6–10 d, was significantly earlier in fuchsia flowers (mean ± SD = 8.3 ± 1.47 d) than in blue flowers (9.6 ± 1.07 d; *F*_1, 59_ = 87.64, *P* < 0.0001). At stigmatic receptivity, one of the anthers could be on its last pollen exposure event. Under natural conditions, more ovules per fruit were found in the blue morph (241.76 ± 4.1) than in those of the fuchsia morph (208.71 ± 5.7; *F*_1, 42_ = 22.31, *P* < 0.0001).

### Nectar standing crops

Nectar standing crop varied significantly between hybrid flower color morphs (nectar volume: *F*_1, 276_ = 10.22, *P* = 0.0016; amount of sugar: *F*_1, 276_ = 8.17, *P* = 0.0046; Suppl. Table S2) and throughout the day (nectar volume: *F*_2, 276_ = 39.07, *P* < 0.0001; amount of sugar: *F*_2, 276_ = 57.48, *P* < 0.0001; Supp. Table S2), but the color morph × time-of-day interaction was not statistically significant (nectar volume: *F*_2, 276_ = 1.24, *P* = 0.29; amount of sugar: *F*_2, 276_ = 1.07, *P* = 0.34). On average, more nectar (volume) was accumulated in fuchsia flowers than in blue flowers, but nectar accumulated in fuchsia flowers was more dilute than blue flowers (nectar volume, *F*_1, 80_ = 5.66, *P* = 0.019; amount of sugar, *F*_1, 80_ = 2.06, *P* = 0.155; Supp. Table S2 and Suppl. Fig. S1).

### Floral visitors

**Figure 1:**
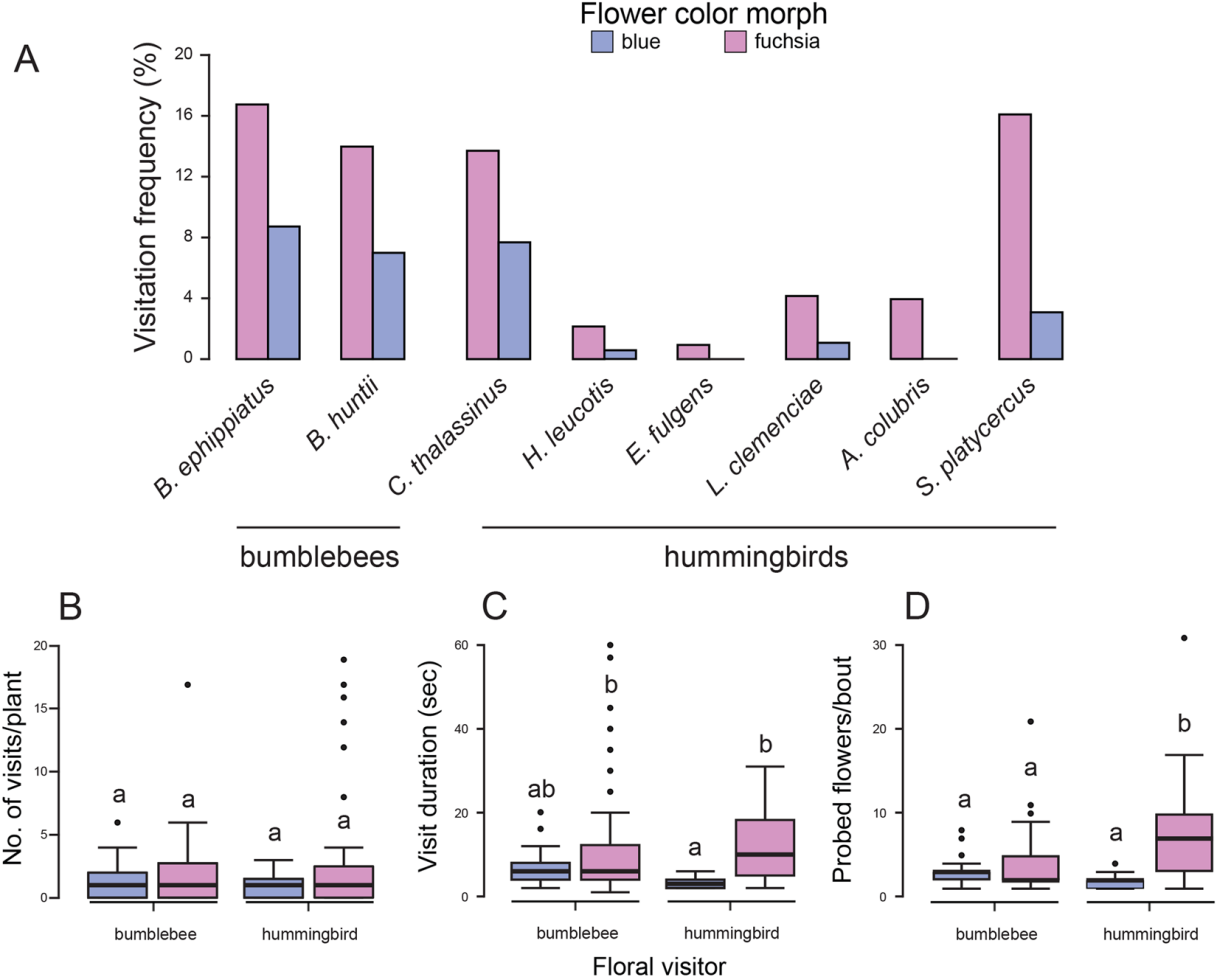
Visitation rate of bumblebees and hummingbirds on *Penstemon* blue and fuchsia flowers. (**A**) visitation frequency (%), (**B**) number of visits per plant (foraging bouts), (**C**) visit duration to focal plant, (**D**) number of flowers probed per foraging bout. The figure was generated using ggplot2 in R (https://cran.r-project.org/web/packages/ggplot2/index.html) and edited in Adobe Illustrator CS6 v16.0.0 (Adobe Systems, Inc.).

A total of 284 visits were registered during our observations, 133 (46.8%) from two bumblebee species (*Bombus ephippiatus* and *Bombus huntii*) and 151 (53.2%) from six hummingbird species (*Colibri thalassinus*, *Eugenes fulgens*, *Lampornis clemenciae*, *Hylocharis leucotis*, *Archilochus colubris*, *Selasphorus platycercus*). The blue morph received fewer visits (25.4%) than the fuchsia morph (74.6%) by all pollinator taxa. Hummingbirds and bumblebees visited more the fuchsia morph (82.1% of all hummingbird visits, 66.2% of all bumblebee visits) than the blue morph (17.9% of all hummingbird visits, 33.8% of all bumblebee visits). The most common floral visitor of the fuchsia morph was *B. ephippiatus* (16.9% of all pollinator taxa), followed by territorial hummingbirds *S. platycercus* (16.1%), and *B. ephippiatus* was the most common floral visitor of the blue morph (8.8% of all pollinator taxa) followed by *C. thalassinus* (7.7%; Fig. 1A).

The time elapsed until a bumblebee or a hummingbird visited either color morph was statistically similar (blue: χ^2^ = 0.3, df = 1, *P* = 0.61; fuchsia: χ^2^ = 3.4, df = 1, *P* = 0.06). However, fuchsia morph individuals received about two more visits than those of the blue morph (mean ± SD, fuchsia = 2.62 ± 4.2, blue = 1.14 ± 1.2 visits; color morph: *F*_1, 132_ = 7.57, *P* = 0.0067), particularly before noon (time-of-day: *F*_2, 132_ = 5.28, *P* = 0.0061; Fig. 1B). Duration of foraging visits varied significantly between pollinator types, depending on the color morph (floral visitor × color morph interaction: *F*_1, 272_ = 4.88, *P* = 0.0279). Bumblebees made longer visits on the blue morph individuals (mean ± SD, 6.5 ± 3.5 sec/visit) than hummingbirds (3.1 ± 1.2 sec/visit), whereas both bumblebees and hummingbirds spent more time on fuchsia morph individuals (10.8 ± 12.1 sec/visit, 11.9 ± 8.1 sec/visit, respectively; Fig. 1C). Lastly, hummingbirds visited about two times more flowers per foraging bout than bumblebees did in the fuchsia morph (mean ± SD, hummingbirds = 6.2 ± 0.45 flowers visited per bout, bumblebees, 3.5 ± 0.44) and more than two times when they or the bumblebees visited the blue morph (floral visitor × color morph interaction: *F*_1, 272_ = 14.08, *P* = 0.0002; Fig. 1D).

### Flower color measurement

**Figure 2:**
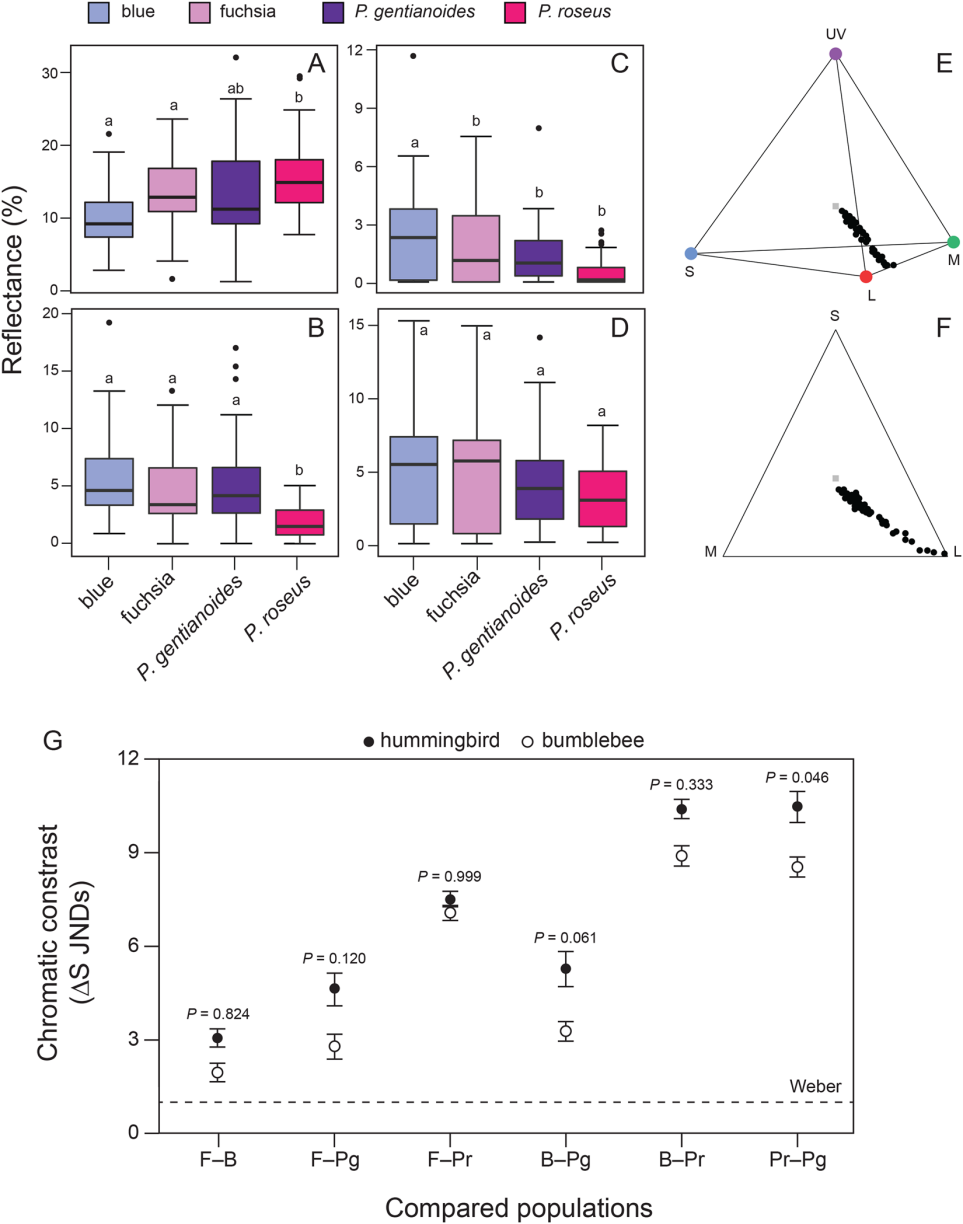
Receiver-centric variables (chroma) of a tetrahedral color space analysis of blue, fuchsia, *Penstemon gentianoides*, and *P. roseus*. (**A**) red, (**B**) blue, (**C**) ultraviolet, (**D**) green. Plot of variables using a sensory based analysis indicating the location of each point in a tetrahedral color space for avian vision (**E**) and bi-dimensional for bee vision (**F**) of blue and fuchsia flowers, with black dots corresponding to individual observations and grey dot represents the mean value. (**G**) Plot showing chromatic contrasts against the background of fuchsia, blue, *P. gentianoides*, and *P. roseus* flowers for hummingbirds (closed circles) and bumblebees (open circles) color perception. Data are color distances (in units of just noticeable differences, JNDs) by color contrast (y-axis) for six contrasts. The dotted horizontal lines indicates JND = 1 (Weber), above which the pair of color contrast is considered to be distinguished by bumblebees and hummingbirds. Points and error bars indicate mean ± standard error chromatic distances between different species and hybrid flower color morphs. *Significance at the 0.05 level, *t*-test. The plots in figure were generated using ggplot2 in R (https://cran.r-project.org/web/packages/ggplot2/index.html) and edited in Adobe Illustrator CS6 v16.0.0 (Adobe Systems, Inc.).

Spectral reflectance in the red, blue, ultraviolet, and green wavelength ranges varied between hybrid flower color morphs and between species. Corollas of *P. roseus* flowers reflected more in the red wavelength range than those of either color morph (*F*_3, 154_ = 8.33, *P* < 0.0001; Fig. 2A). Moreover, *P. roseus* flowers reflected less in the blue wavelength than those of either color morph or *P. gentianoides* flowers (*F*_3, 154_ = 11.60, *P* < 0.0001; Fig. 2B). When we looked at UV wavelength values, blue flowers reflected more UV wavelength than those of the other groups of flowers (*F*_3, 154_ = 6.44, *P* = 0.0004; Fig. 2C), whereas for the green wavelength we found no differences among groups of flowers (*F*_3, 154_ = 2.41, *P* = 0.0689; Fig. 2D). Further, we found that the greater percentage of relative stimulation from the floral reflectance was associated with the receiving cones of long length (L) waves, for both visitors (hummingbird: *F*_2, 234_ = 176.24, *P* < 0.0001; bumblebee: *F*_2, 234_ = 176.2, *P* < 0.0001; Suppl. Table S3). However, discrimination between flowers was due to reception of medium waves (M) in hummingbirds (Fig. 2E) and medium-to-short waves (M and S) in bumblebees (Fig. 2F). We also found that the angle of the visual signal differed in each group (X: *F*_3, 75_ = 40.52, *P* < 0.0001, Y: *F*_3, 75_ = 6.19, *P* = 0.0008, h.theta: *F*_3, 75_ = 7.03, *P* = 0.0003 for hummingbirds; X: *F*_3, 75_ = 27.46, *P* < 0.0001; Y: *F*_3, 75_ = 38.42, *P* = 0.0001; h.theta: *F*_3, 75_ = 50.44, *P* < 0.0001 for bumblebees; Suppl. Table S3). Likewise, the h.phi angle on the Z axis for hummingbirds showed a negative angular displacement (Suppl. Table S3), which confirms a visual stimulation from medium waves (M), in which the blue and *P. roseus* flowers differed from fuchsia and *P. gentianoides* flowers (Z: *F*_3, 75_ = 65.09, *P* < 0.0001; h.phi: *F*_3, 75_ = 6.38, *P* = 0.0006).

We found 5.5 ± 1.17 JNDs (just noticeable differences) above the discrimination criteria for bee vision and 7.16 ± 1.32 JNDs for hummingbirds (*F*_3, 75_ = 179.41, *P* < 0.0001; Fig. 2G). When we looked at flower detectability against the background, we did find that blue flowers are more easily detected than fuchsia flowers considering chromatic contrasts (8.00 ± 1.34 vs. 4.4 ± 0.88 JNDs, *t* = 3.05, *P* = 0.028) and distinguished by pollinator type (hummingbird: fuchsia = 1.75 ± 0.08 JNDs, blue = 0.8 ± 0.03 JNDs, *t* = 10.25, *P* = 0.001; bumblebee: fuchsia = 0.98 ± 0.04 JNDs, blue = 0.39 ± 0.01 JNDs, *t* = 12.98, *P* = 0.001). And blue flowers were easily detected against *P. roseus* as compared to fuchsia flowers, and *P. roseus* flowers against *P. gentianoides* as compared to fuchsia flowers for hummingbirds and bumblebees (Fig. 2G). However, we found no discrimination between blue and fuchsia flowers with *P. gentianoides* (Fig. 2G). Further, JNDs varied by color contrast, floral visitor and their interaction (color contrast: *F*_5, 4668_ = 112.83, *P* < 0.0001; floral visitor: *F*_1, 4668_ = 31.73, *P* < 0.0001; color contrast × floral visitor interaction: *F*_5, 4668_ = 2.32, *P* = 0.040). However, we only found color discrimination by hummingbirds and bumblebees in the *P. roseus*/*P. gentianoides* contrast (*P* = 0.046; Fig. 2G). Finally, we found a higher spectral purity (rA) received by hummingbirds in *P. roseus* (*F*_3, 75_ = 62.54, *P* < 0.0001; Suppl. Table S3), favoring detection and discrimination by hummingbirds. Although this measure was not generated for bumblebees because of their two-dimensional visual perception, we found a similar trend for the chromatic value (r), being higher for *P. roseus* (*F*_3, 75_ = 30.18, *P* = 0.0001; Suppl. Table S3).

### Pollinator effectiveness

We found fruit set differences between hybrid flower color morphs under natural conditions (blue = 14.35 ± 0.9 fruits/plant, fuchsia = 20.12 ± 1.21; *F*_1, 39_ = 14.59, *P* = 0.00026). On average, fuchsia flowers produced wider and heavier fruits and more seeds per fruit than blue flowers (fruit length, *F*_1, 37_ = 0.81, *P* = 0.36; fruit width, *F*_1, 37_ = 24.88, *P* < 0.0001; fruit weight, *F*_1, 37_ = 74.18, *P* < 0.0001; number of seeds per fruit, *F*_1, 37_ = 66.43, *P* < 0.0001; Suppl. Fig. S2).

**Figure 3:**
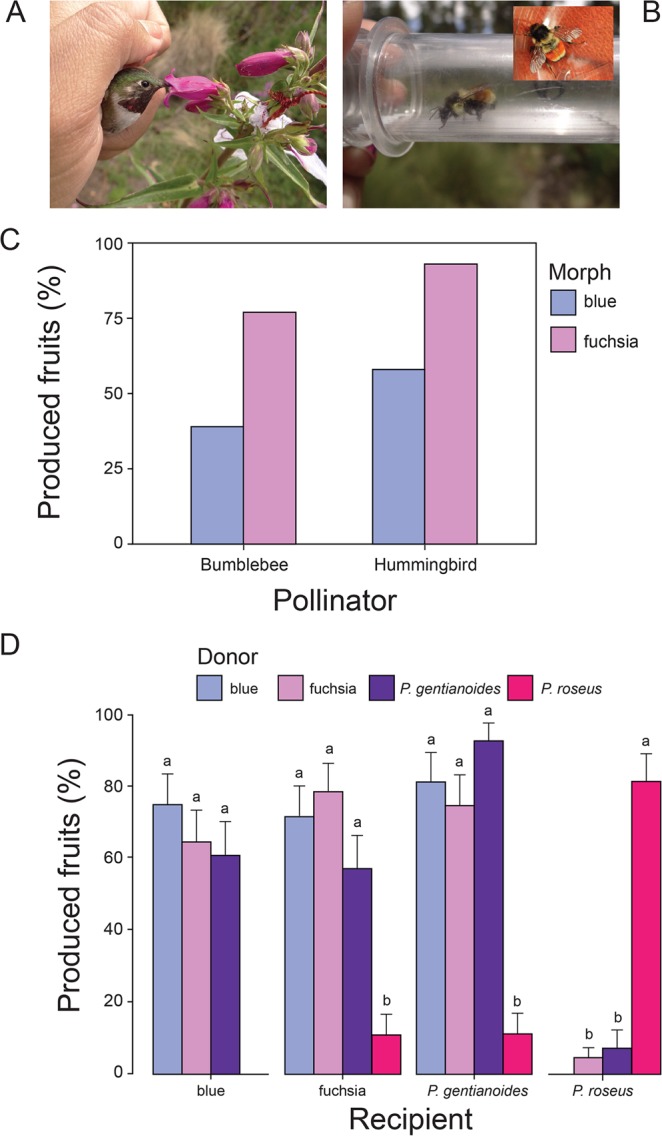
Main pollinators of *Penstemon* blue and fuchsia flowers. (**A**) *Selasphorus platycercus* (by C. Lara); (**B**) *Bombus ephippiatus* (by J. Cardona); (**C**) fruit production and effectiveness of bumblebees and hummingbirds as pollinators of flowers within each color morph; (**D**) fruit production after reciprocal crosses between species, *Penstemon gentianoides* and *P. roseus*, and between hybrid flower color morphs. Data (mean ± SE) with the same superscript letters are not significantly different between groups (*P* < 0.05). Tukey post hoc tests: *P* < 0.05 for all pairwise comparisons. The plots in figure were generated using ggplot2 in R (https://cran.r-project.org/web/packages/ggplot2/index.html) and edited in Adobe Illustrator CS6 v16.0.0 (Adobe Systems, Inc.).

We found a higher probability to fruit formation when flowers were pollinated by hummingbirds (75.8%) than flowers pollinated by bumblebees (58.1%; χ^2^ = 5.29, df = 1, *P* = 0.021; Fig. 3A–C). Moreover, fuchsia flowers initiated about two times more fruits (85.4%) than blue flowers did (48.3%; χ^2^ = 20.98, df = 1, *P* < 0.0001), and the color morph × pollination type interaction was not significant (χ^2^ = 0.45, df = 1, *P* = 0.498). When pollinated by hummingbirds, we found that fuchsia flowers produced longer and heavier fruits with more seeds than the blue flowers they pollinated, whereas blue flowers produced longer fruits with more seeds than the fuchsia flowers when pollinated by bumblebees (Table 1; Suppl. Fig. S2).

**Table 1:** Pollinator and color morph effects on fruit size and number of seeds produced by bumblebee- and hummingbird-pollinated flowers that set fruit of *Penstemon* blue and fuchsia morphs.

Variable	Factor	SS	*df*	*F*	*P*
Fruit length	Morph	0.435	1	0.441	0.505
	Pollinator	33.199	1	33.658	<0.0001
	Morph × pollinator	19.026	1	19.289	0.00003
	Residual	77.921	79		
Fruit width	Morph	0.002	1	0.0059	0.939
	Pollinator	0.488	1	1.361	0.247
	Morph × pollinator	3.245	1	9.037	0.0035
	Residual	28.369	79		
Fruit weight	Morph	0.021	1	27.781	<0.0001
	Pollinator	0.036	1	46.202	<0.0001
	Morph × pollinator	0.018	1	22.936	<0.0001
	Residual	0.061	79		
Number of seeds	Morph	7718	1	10.888	0.0015
	Pollinator	4801	1	6.773	0.011
	Morph × pollinator	12457	1	17.574	<0.0001
	Residual	55290	78		

Effects were modelled using a generalized linear model (GLM) with Gaussian error distribution and logarithmic link function, and flower color morph (blue or fuchsia) and pollinator type (bumblebee or hummingbird) treated as fixed effects and measures as continuous response variables.

### Reproductive isolation

Seed set was affected by pollination treatment (donor effect, χ^2^ = 47.91, df = 3, *P* < 0.0001; recipient effect, χ^2^ = 34.26, df = 3, *P* < 0.0001) and the donor × recipient interaction was statistically significant (χ^2^ = 175.54, df = 9, *P* < 0.0001). When *P. roseus* was the recipient plant, we found a greater proportion of fruits after intraspecific crosses (>80%) than either backcrosses (<15%) or interspecific crosses (zero; Fig. 3D). For *P. gentianoides* recipients, more fruits were produced when pollen was intraspecific (>90%) but similar to that in backcrosses (c. 80%) and higher to that in the interspecific treatment (15%; Fig. 3D). On hybrid flower color morph plants, fruit set across color morphs or backcrosses with *P. gentianoides* (>50%) were similar, but higher than that after color morph crosses with *P. roseus* (<15%; Fig. 3D). Finally, we found that fruit size values and seed numbers for flowers that set fruit were generally higher when blue, fuchsia and *P. gentianoides* plants were recipient than in *P. roseus* recipient plants (Table 2; Suppl. Fig. S3).

**Table 2:** Effects of species and color morphs on fruit size and number of seeds produced by manually-pollinated flowers that set fruit of *P. gentianoides*, *P. roseus* and blue and fuchsia morphs.

Variable	Factor	SS	*df*	*F*	*P*
Fruit length	Donor	6.249	3	5.039	0.0022
	Receptor	247.389	3	199.500	<0.0001
	Donor × Receptor	24.158	7	8.349	<0.0001
	Residual	83.496	202		
Fruit width	Donor	6.632	3	3.305	0.021
	Receptor	46.565	3	33.231	<0.0001
	Donor × Receptor	3.942	7	1.206	0.301
	Residual	94.350	202		
Fruit weight	Donor	0.053	3	36.013	<0.0001
	Receptor	0.115	3	77.952	<0.0001
	Donor × Receptor	0.110	7	31.936	<0.0001
	Residual	0.099	202		
Number of seeds	Donor	9173	3	6.907	0.00019
	Receptor	94112	3	70.871	<0.0001
	Donor × Receptor	13061	7	4.215	0.00023
	Residual	89414	202		

Effects were modelled using a generalized linear model (GLM) with Gaussian error distribution and logarithmic link function, and donor and receptor treated as fixed effects and measures as continuous response variables.

Hybrid flower color morphs varied in flower morphology and nectar traits when compared with parental species (Suppl. Fig. S4). We found that fuchsia and blue flowers were significantly larger (corolla length: *F*_3, 574_ = 820.28, *P* < 0.0001; corolla entrance width: *F*_3, 574_ = 124.96, *P* < 0.0001) than those of both parental species, and stamen (*F*_3, 574_ = 787.62, *P* < 0.0001) and style (*F*_3, 574_ = 156.79, *P* < 0.0001) were intermediate in length between *P. gentianoides* and *P. roseus* (Suppl. Fig. S4). Stamen and style lengths were significantly larger in *P. roseus* than either color morphs or *P. gentianoides*, with lower values in *P. gentianoides* (Suppl. Fig. S4). For nectar traits, we found that the volume of nectar was significantly larger in *P. roseus* flowers than in *P. gentianoides* and both parental species produced more nectar than either color morph (*F*_3, 488_ = 100.43, *P* < 0.0001; Suppl. Fig. S4). However, more sugar was found in the nectar of *P. gentianoides* than in *P. roseu*s, blue or fuchsia flowers (*F*_3, 487_ = 33.12, *P* < 0.0001; Suppl. Fig. S4).

Using *RI* estimate *RI*_*4A*_, there were fewer matings than expected by chance in *P. roseus* when crossed with *P. gentianoides*, blue, or fuchsia flowers (*RI*_*4A*_ = 0.84, 1.0 and 0.92, respectively) as compared to all other types of crosses (Suppl. Table S4). These three types of crosses also had higher *RI*_*4C*_ values, which take other factors of ecological isolation into account (i.e., pollinator assemblage as prezygotic barrier), relative to the other types of crosses (Suppl. Table S4).

## Discussion

The two parental species (*Penstemon gentianoides* and *P. roseus*), occuring primarily at different altitudes, produce two hybrid flower color morphs where they come into contact, which creates conditions for speciation. Our study used variation in the hybrid zone to test whether intermediate floral traits deter bumblebees and promotes the evolution of hummingbird pollination. In addition to differences in color (spectral reflectance), the flower morphology, floral display and nectar production patterns differed between fuchsia and blue morphs. These differences influenced the foraging behavior and effectiveness as pollinators of both bumblebees and hummingbirds, which contributed to reproductive isolation between the two hybrid flower color morphs and parental species.

This study is the first to show the existence of two hybrid flower color morphs between two *Penstemon* species with mixed-pollination systems. The two hybrid flower color morphs and *P. roseus* show intermediate floral and nectar traits between bee- and hummingbird-pollination modes, with increased nectar volume (dilute nectar) and higher reproductive isolation of *P. roseus* when crossed with *P. gentianoides*, blue, or fuchsia flowers in a likely pathway to hummingbird pollination. Apart from color differences, previous studies have found that the flower morphology and the sugar content were similar between flower color morphs concomitantly pollinated by bees and hummingbirds^21,51,52^. Our results indicate that *P. roseus* and fuchsia flowers conform with recent evidence for repeated transitions toward increased nectar production in hummingbird-syndrome *Penstemon*^18,46^, and that evolutionary transitions from one syndrome to another require changes in multiple floral traits required for pollination syndrome evolution^16,18,53^.

In this study, flower color hybrids did not present clear morphological distinctions. However, hybrids might not be identified as a parental species based only on morphology. Although the flower color hybrids are not intermediates, they have an unusual morphology when compared to the parental species. Flowers of the two flower color hybrids were longer and had wider mouths than flowers of parental species, where fuchsia flowers were larger, while *P. gentianoides* were shorter and *P. roseus* had narrower mouths. The stamen and style lengths of the flower color hybrids were intermediate between parental species, where *P. roseus* had more exserted stamens and styles, while stamens and styles were more included in *P. gentianoides*. *Penstemon roseus* produced more than two times as much nectar as *P. gentianoides*, while the flower color hybrids produced a similar amount of nectar but less than the parental species. Furthermore, the ultraviolet reflectance of blue morph was higher than the fuchsia morph and the parental species, produced more ovules per flowers and had a reproduction barrier with *P. roseus* but less barrier with the fuchsia morph and *P. gentianoides*, which indicate that the blue morph may be not the intermediate morph. Hybrids need not be intermediate in floral morphology or other traits between the parental species, but instead can be transgressive, or more extreme than either parental species^31,32^. Second generation (F2) hybrids formed by crossing two F1 individuals often exhibit transgressive traits^54^, notably the size-related characters of corolla width and petal width, and nectar volume, and little transgressive segregation in characters related to flower color^4,31,54,55^.

**Figure 4:**
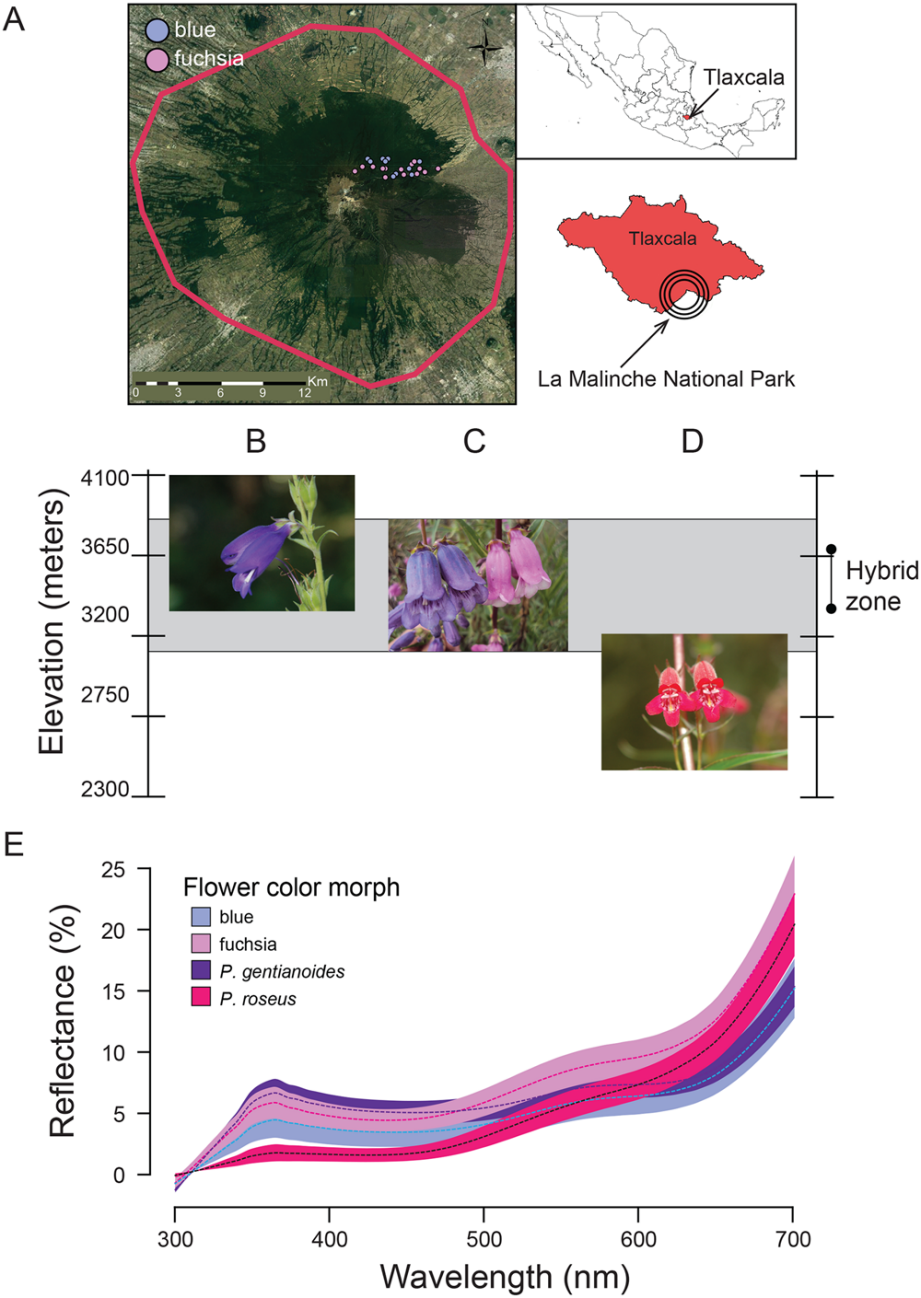
Study site, study species and spectral color reflectance measures of the blue and fuchsia morphs. (**A**) La Malinche National Park (LMNP), Tlaxcala, Mexico. The geographic map was generated with the software ArcGIS Desktop 10.1 (ESRI 2001, Redlands, CA) based on satellite imagery source (https://earth.google.com/web/@19.22446138,-98.03311368,4072.84173853a,26147.38187905d,35y,0h,0t,0r) from Google (Google Maps satellite La Malinche National Park, Tlaxcaka, Mexico; retrieved February 17, 2019). ArcGIS Desktop 10.1 software was downloaded from https://arcgis-sp1-for-desktop-quality-improveme.software.informer.com/. Dots indicate the exact locations of floral patches of the blue and fuchsia morphs. The figure was drawn using Adobe Illustrator CS6 v16.0.0 (Adobe Systems, Inc.). (**B**) *Penstemon gentianoides* (by C. Lara). (**C**) blue (left) and fuchsia (right) morphs (by C. Lara). (**D**) *Penstemon roseus* (by C. Lara). (**E**) spectral color reflectance measures of the parental species and the blue and fuchsia morphs (±SE). The plot was generated using ggplot2 in R (https://cran.r-project.org/web/packages/ggplot2/index.html) and the figure drawn and edited in Adobe Illustrator CS6 v16.0.0 (Adobe Systems, Inc.).

In the sensory-based analysis for bee and bird vision, blue morph had extreme (highest or lowest) values for relative cone stimulation, tetrahedral color space and hue (h.theta, h.phi) and chroma (r and rA) measures as compared to those of the fuchsia morph and parental species (Table S3), but intermediate between the fuchsia morph and parental species in shorter wavelengths (reflectance spectra near 400 nm or 500 nm; Fig. 4E), which allows bees to see UV radiation^26,28^. The fuchsia morph and *P. roseus* were clustered between 600 nm and 700 nm (long wavelength “red” radiation poorly discriminated by bee photoreceptors^26,28^), which is expected for hummingbird-adapted flowers^26^ because of the violet sensitive (VS) color vision of hummingbirds^19^. Bees require more time to find red flowers than blue ones^56^, while hummingbirds seem to detect them equally well. In our study site, blue morph plants are scarce compared to the fuchsia morph and parental species, and despite the higher availability of nectar in its flowers, they received fewer visits by both pollinator types. Nonetheless, the low visitation rates by both pollinator types seem to be sufficient to maintain the population of the blue morph in the hybrid zone. Thus, hybridization between *P. roseus* and *P. gentianoides* may result in the uncoupling of the species-specific combinations of flower color and nectar production affecting the ability of hummingbirds and bees to discriminate against hybrid individuals whose flowers offer very little nectar but resemble *P. roseus* or *P. gentianoides* in color. Further research (e.g., QTL mapping) is needed to assess whether flowers of hybrids are intermediate or transgressive in phenotype.

The coexistence of the hybrid flower color morphs on the same location, and nearby to the putative parental species, creates the conditions that influence pollinator foraging decisions affecting gene flow among them. At the study site, hybrid flower color morphs followed a unimodal flowering pattern, which overlaps between morphs and the flowering season of *P. gentianoides*^47^. The temporal separation of the sexual function reduces self-pollination and the extended male phase slows down removal of pollen by visitors and increase male reproductive success^36,48^. In our samples, numbers of ovules per flower in each color morph are similar to those in *P. gentianoides*^47^ but fewer ovules per flower were reported in *P. roseus*^48^. Although blue flowers produce more ovules per flower, fuchsia flowers produced wider and heavier fruits and more seeds per fruit under natural conditions. This suggests limitation on the resources in plants with blue flowers to mature seeds or that stigmas on these natural pollinations were provided with insufficient and/or low quality pollen by lousy pollinators^48^. Unfortunately, our study did not address male components of pollination effectiveness (e.g., variation among visitor taxa in pollen dispersal). In flowers, which adapted to large pollinator visits like hummingbirds, it is expected to find removal-enhanced replenishment of nectar^57,58^. Multiple nectar removals by flower visitors typically enhance the volume produced by flowers, but repeated nectar removal causes declines in the amount of sugar produced^39,57^. Although replenishment of repeatedly removed nectar incurs a metabolic cost that can lower female reproduction^48,59,60^, replenishing dilute nectar in flowers with continuous visitation may compensate for this cost^58^. Removal-enhanced replenishment of nectar is well documented in several *Penstemon* species^39,46,47^. However, future comparisons among the flower types in our study should test (1) whether plants mainly visited by bumblebees or by hummingbirds expend additional energy to replenish removed nectar, (2) plants with intermediate morphologies and mixed-pollination systems respond similarly to those adapted to bumblebee or hummingbird pollination, and (3) whether nectar replenishment and pollination intensity jointly affect seed production^48^.

We found floral trait differences between hybrid flower color morphs, and differences with respect to either parent *P. gentianoides* and *P. roseus*, where hybrids resemble one of the parental species more closely. However, these results differ from what was previously reported in the hybrid zones between *P. newberryi* and *P. davidsonii* along an elevational gradient in the Sierra Nevada of California, where hybrids are more abundant than either parent at intermediate elevations^61^. It was found that plants varied phenotypically in a clinal pattern along the elevational gradient, suggesting hybridization beyond the F1 generation. The differences in the pollinator community composition between these *Penstemon* species and hybrids may promote ecological isolation. However, the most common pollinator species was shared by parent species, which might be contributing to hybrid formation^61^.

Despite flower size differences between fuchsia and blue morphs, the higher accumulated nectar throughout the day in fuchsia flowers resulted in smaller nectar standing crops as compared to blue flowers, a pattern similar to that in *P. gentianoides*^47^. There are three potential explanations for this pattern: (1) reduced competition for pollinators where plants overlap in their flowering time because of the differences between bee and hummingbird flowers traits reduce competition for food among the floral visitors^62^; (2) the response of plants to nectar removal in which the increased frequency of visitation would stimulate nectar replenishment and reward overcompensation in fuchsia flowers^48,63^; and (3) the observed morph differences in nectar production might be simply a physiological response to the varied environmental conditions, particularly humidity^64^. Unfortunately, this factor was not controlled, because nectar measurements were made on different days and possibly under different conditions, and the amount of nectar removed by floral visitors was not quantified for individual flowers. Thus, the genetic basis to environmental differences associated with morph color divergence need to be investigated to assess these hypotheses.

We showed that *P. gentianoides*, *P. roseus*, and the hybrid flower color morphs are distinguishable by bumblebees and hummingbirds. Blue and *P. gentianoides* flowers reflect short-wavelength-rich ultraviolet, violet or blue light, whereas fuchsia and *P. roseus* flowers appear to reflect longer wavelengths of reddish colors. As expected, hummingbird’s visual system analysis showed higher color discrimination values, possibly due to better perception and detection of longer wavelength reddish signals^21,22,65,66^. These results coincide with the idea that flower color detectability associated with the red color blindness of bees is the factor driving the different levels of visitation specialization in red-reflecting flowers^21,22,30^. The three- and tetra-dimensional representation for the visual system of pollinators indicated that stimulation from flower reflectance allows pollinator types to discern between flowers, more strongly for medium wavelengths in hummingbirds and short-to-medium wavelengths in bumblebees. However, color discrimination differences between bumblebees and hummingbirds were only statistically significant for the *P. roseus*/*P. gentianoides* contrast after post-hoc mean comparisons. Because the higher spectral purity of *P. roseus* flowers, and the higher JND values for hummingbird discrimination in all chromatic contrasts as compared to those for bumblebees, gene flow from *P. roseus* into the *P. gentianoides* and the hybrid zone might be promoted by hummingbirds, as previously documented in an *Ipomopsis* hybrid zone^32^. Our results imply that species that undergo bee-to-bird transitions in pollination evolve not merely ‘anti-bee’ floral colors, but colors that emphasize a narrow region of the electromagnetic spectrum providing the best opportunity to be distinguished by birds from competing bird-pollinated flowers.

Also, we showed that phenotypic differences between parental species and hybrid flower color morphs influenced the foraging behavior and pollinator effectiveness of bumblebees and hummingbirds, as hybrid formation is determined by both factors^31^. Although flower visitation was not evaluated simultaneously among all groups of plants, previous reports indicate that both pollinator types play an important role in the pollination of both parental species^46,47^. The pollinator community composition also varied with altitude at LMNP, which coincide with previous research^61^, and the pollinator community of *P. roseus* differed from those in *P. gentianoides* and color morphs in the number of hummingbird species and their foraging frequency^46,47^. Bumblebees and hummingbirds visited more frequently the fuchsia flowers. Thus, fuchsia flowers that accumulate more nectar (i.e., dilute nectar) had smaller standing crops presumably because they were more visited than blue flowers.

Considering the patterns of pollinator visitation, our findings of higher visitation frequency for fuchsia flowers coincide with the pollinator effectiveness experiments (see below). Although we cannot fully exclude the possibility of specialization towards hummingbird pollination, parental species and hybrids seem to be equally fit and therefore coexist in the center of the studied gradient. Thus, while variation in floral traits, particularly in the fuchsia morph, is directed to the attraction of an assemblage that transcends hymenoptera to a broader community of pollinators, which includes hummingbirds, its mixed pollination system might be maintained by hybridization^45^. Here, we showed that bumblebees made shorter visits and probed fewer flowers in the fuchsia morph compared to hummingbirds. These results suggest that red reflection of the fuchsia flowers can be acting as a sensorial barrier to bumblebees along with other synergistic interactions among floral traits and nectar availability that mediate pollinator efficiency and resource partitioning between bumblebees and hummingbirds^2,38,50,56^.

We found that hummingbirds pollinate most effectively fuchsia flowers than they do blue flowers, which seems predictable for hummingbird-pollinated *Penstemon* flowers^35^. The discrimination of floral color related to pollination efficiency of bumblebees and hummingbirds has been previously evidenced in other systems such as *Pedicularis*^67^, *Lobelia*^68^ and *Mimulus*^55^. Interestingly, we found that bumblebees and the most common hummingbird species (*S. platycercus*) were the most frequent visitors of fuchsia flowers and the opposite usually in blue flowers, so the importance of both pollinators were flipped between the two morphs. However, pollinator sharing might be contributing to directional hybrid formation because *S. platycercus* visited *P. gentianoides* and the hybrids most frequently, whereas *P. roseus* was most visited by resident *Hylocharis leucotis* and *Eugenes fulgens*^46,48^. Overall, as observed in the present study, pollinators do sequentially visit the various hybrid and parental classes that flowered at the same time, but they do so with marked preferences. Thus, the pollinators are likely acting as a strong yet incomplete barrier to introgressive hybridization^31,61^.

As shown by previous research, fruit production in *P. gentianoides* is higher when flowers are pollinated by the most common floral visitors (bumblebees), as compared to those pollinated by the most common hummingbird species *S. platycercus*^47^. And resident hummingbirds were the main floral visitors of *P. roseus*. However, bumblebees and butterflies also visited its flowers and pollinator effectiveness experiments were not conducted for *P. roseus*^46,48^. Although pollen transfer efficiency by hummingbirds and bumblebees for fuchsia and blue flowers are not available, experimental data from previous research indicate higher pollen transfer efficiency by hummingbirds in *Penstemon* species with the hummingbird-pollination syndrome as compared to those with the bee-pollination syndrome^38^. Also, bumblebees were less efficient and effective pollinators of *P. gentianoides* were altered when its flowers were modified simulating hummingbird-pollination characteristics^50^. Thus, because differences between bumblebees and hummingbirds in color discrimination were only clear for the *P. roseus*/*P. gentianoides* contrast, reproductive differences between flower color morphs would be produced by differential pollen transfer (pollen quality and quantity) due to their foraging behavior differences and the ‘pro-bird’ characteristics of the fuchsia flowers^35,38,40^.

We found that fruit set was generally higher among conspecific crosses than in backcrosses or heterospecific crosses, suggesting pre-pollination and post-pollination barriers to reproductive isolation between hybrid flower color morphs and parental species. Nonetheless, there was a high amount of asymmetry in the success of crosses, in that *P. roseus* can really only be crossed with itself, while the other morphs/species can be crossed with each other. One reason for the asymmetrical success of crosses might relate to the differences in reliance on different pollinators and, as discussed above, patterns of pollinator sharing that ease reproductive (pre-zygotic) isolation to occur between *P. roseus* and the other morphs/species^55^. Considering other factors of ecological isolation that affect co-occurrence, we found that reproductive isolation (*RI*) values for isolation by pollinators (*RI*_*4C*_) were higher between *P. roseus* and *P. gentianoides* and the two color morphs, as compared to the other crosses evaluated. Additionally, the absolute and relative cumulative strength values of *RI* indicate that pollinator behavior is currently contributing the most to maintenance of reproductive isolation between parental species and between parental species and hybrid flower color morphs. Because the species are not closely related^69,70^, post-pollination barriers to reproductive isolation and adaptation to different environments, in which elevational and habitat differences maintain species as different entities^71,72^, are probably acting to reproductive isolation between *P. gentianoides* and *P. roseus* (*RI*_4A_ = 0.8). Kimball and collaborators^73^ measured reproductive isolation by crossing two closely related parent species of *Penstemon* (*P. davidsonii* and *P. newberryi*) and naturally occurring hybrids in a reciprocal transplant experiment. They found that interspecific crosses produced, on average, as many seeds as conspecific crosses, and hybrid performance were also equal to or greater than parents in all environments, suggesting a lack of endogenous selection. However, parent species and reciprocal F_1_ hybrids differed in many traits measured and the hybrid with the native cytoplasm had a higher survival rate, highlighting local adaptation to different elevations and the importance of exogenous factors. Thus, a reciprocal transplant experiment in which fitness is assessed in multiple habitats is needed to distinguish between endogenous and exogenous reproductive isolation between the parental species and their hybrids.

## Conclusions

We provide evidence of how intermediate or transgressive floral characters might have evolved under hummingbird mediated pollination, in which fuchsia and blue flowers have evolved color signals at spectral positions of maximal pollinator color discrimination. The morphs evaluated represent populations whose peaks of maximum flowering overlap, but exhibit differences in their population density, mechanisms of floral anthesis, floral morphology, flower color and nectar production. The evaluated floral and nectar traits of the hybrid flower color morphs seem to be intermediates between the parental species, transgressive, or more extreme than either parental species, not limiting so that both bumblebees and hummingbirds can access the resource. However, considering the role of each pollinator type, it is evident that hummingbirds are the most effective group within this mutualistic interaction, and those that probably promote the maintenance of a potential hybrid zone. The flowering patterns between sympatric *Penstemon* species must be the subject of future studies, covering environmental conditions and antagonistic interactions across space and time.

## Methods

### Study site

Research was conducted at the La Malinche National Park (LMNP), Tlaxcala, Mexico (19°15.205′ N, 098°02.080′ W; elevation 3,400–3,900 m; Fig. 4A). The LMNP is located in a temperate montane forest with an old-growth fir forest (*Abies religiosa*) frequently mixed with *Pinus hartwegii* above 3,500 m, and hosts a diversity of plants that rely on hummingbirds for successful reproduction^74^.

### Study species

*Penstemon gentianoides* (Plantaginaceae) is an herbaceous perennial plant distributed in the major volcanic peaks of the Trans-Mexican Volcanic Belt (TMVB), and southward into Chiapas to Guatemala^69,75^. The species is found at middle and high elevations (3,000–4,200 m), from pine forests typically in open or disturbed sites to slightly above timberline associated with the alpine grasslands^69,75^. At the LMNP, *P. gentianoides* is found in open areas of pine and fir forests, ranging from 3,000 to 3,900 m^47^. Individuals (0.5–1.5 m high) bear 15–25 paniculate inflorescences, each with 2–4 pendant flowers from terminal branching stems opening per day, and **∼**90 floral buds may eventually reach the flower stage during the blooming season (4 months), which extends from July to November^47,74^. *Penstemon gentianoides* flowers (Fig. 4B–D) are protandrous and long-lived^47^, with 8-d male phase (staminate) flowers, followed by the **∼**1–7 d female phase (pistillate phase). Flowers are blue, violet or purple and vestibular flowers abruptly expanding into a broadly inflated throat and a prominent lower lip with anthers and stigmas nearly included^35,47^. However, in contrast to these traits that are often linked to bee pollination, nectar is abundant and dilute at morning hours, which are traits linked to hummingbird pollination^47^. *Penstemon roseus* is an herbaceous perennial plant distributed along the TMVB (Fig. 4D)^69,75^. This species is found at lower elevations (1,800–3,500 m) associated with oak and pine-oak forests and typically in open or disturbed sites^69,75^. Individuals (0.4–1.2 m high) bear 10–20 paniculate inflorescences, each with 2–4 pendant flowers from terminal branching stems opening per day, and **∼**80 floral buds may eventually reach the flower stage during the blooming season (4 months), which extends from July to December^46,48^. *Penstemon roseus* flowers are protandrous and long-lived^46^, with 2-d male phase (staminate) flowers, followed by the **∼**2 d female phase (pistillate phase). Flowers are pink to red and tubular, intermediate between hummingbird- and bee-adapted *Penstemon* flowers. The corolla tube with a globular shape, enlarged ventral lobes and a white throat, contains abundant and dilute nectar^46,48^.

The community assemblage of pollinators varies locally. At 2,900 m, large hummingbirds (*Colibri thalassinus*, *Eugenes fulgens*, *Lampornis clemenciae*) defend intensively the floral patches of *P. gentianoides*^74^, but bumblebees (*Bombus ephippiatus*, *B. huntii* and *B. weisi*) and four smaller hummingbird species (*Hylocharis leucotis*, *Archilochus colubris*, *Selasphorus platycercus*, *Atthis heloisa*) are its main floral visitors slightly above timberline^47^. The long-distance seasonal migrants *S. platycercus* and *A. colubris* are the most frequent (30.3% and 11.3% of all visits, respectively), followed by resident *H. leucotis* (2.9%) and *A. heloisa* (1.2%), and bumblebees and bees (*Apis mellifera*) account for 51.8% and 2.4% of all visits, respectively; hummingbirds probe more flowers per foraging bout than bumblebees^47^. While flowers experimentally pollinated by *B. ephippiatus* produce more fruits than those pollinated by *S. platycercus* (79% and 46%, respectively), single-visit efficiency measures (number of seeds per fruit) derived from bumblebee or hummingbird pollination were similar in *P. gentianoides* (for more details on the experimental set-up see Salas-Arcos *et al*.^47^). Eight hummingbird species visit *P. roseus* flowers at the LMNP: *Colibri thalassinus*, *Hylocharis leucotis*, *Lampornis clemenciae*, *Eugenes fulgens*, *Archilochus colubris*, *Selasphorus rufus*, *S. platycercus*, and *S. sasin*^46^. The most frequent hummingbird is the resident *H. leucotis* (46–65% of all visits), and bumblebees and butterflies are uncommon floral visitors at the LMNP^48^.

The populations of *P. gentianoides* and *P. roseus*, circumscribed to different clades^69,70^, are self-compatible (∼25–28% and 50%, respectively) but outcrossed flowers produce approximately two times more seeds than self-pollinated flowers^46,47^. The distributions of these species overlap forming a hybrid zone when growing in sympatry at the lower elevational range of *P. gentianoides* (Fig. 4A). At the hybrid zone, numerous individuals are similar in floral morphology to *P. gentianoides*, but the color of their corollas is different and likely the product of hybridization with *P. roseus*; no other *Penstemon* species is found at the LMNP^69^. This naturally occurring hybrid zone barely overlaps with a population of *P. roseus* at lower elevation. Individuals present intraspecific floral color variation (Fig. 4C), ranging from blue to fuchsia (hereafter referred as blue and fuchsia morphs) in which reflectance is higher in fuchsia flowers (*F*_1, 37_ = 26.16, *P* < 0.001, see details of color measurements below). Fuchsia individuals open more flowers (mean ± SD = 62.9 ± 23.7 cm tall, *N* = 30) than those of the blue morph (71.7 ± 19.1 cm tall, *N* = 43), forming patches between 3,731–3,922 m and those of the blue morph growing isolated between 3,703–3,738 m. Within the study area (Fig. 1A), the fuchsia:blue morph ratio is 3:1 (213 fuchsia individuals and 68 blue individuals in two 50 × 50 m plots spaced at least 150 m apart). The hybrid flower color morphs flower from July to November, with a flowering peak in August. Seasonal reproductive isolation is unimportant because flowering time of the two hybrid flower color morphs overlap almost completely. Within plants, flowers open asynchronously (30% per inflorescence) and are protandrous.

### Floral biology

During the blooming season (July–November 2016), we selected 67 plants of each color morph, measured 2–3 fully developed flowers from each of the plants (*N* = 268 flowers), noting whether sexual organs were exserted, and measured the flower color (see below). Corolla tube length (distance from the base of the corolla to the corners of the corolla mouth), corolla-entrance width, corolla-entrance height, and filament and style lengths were measured with a digital calliper (error: 0.01 mm).

In September 2016, we daily inspected 40-tagged buds growing on 20 additional plants of each hybrid flower color morph until wilting to evaluate floral longevity and length of each sexual phase. We assessed stigma receptivity of 40 flowers per color morph over 12 days after flower opening by submerging stigmas into a 32% hydrogen peroxide solution and using the presence of bubbling on the stigma to infer receptivity^76^. We also counted the total number of ovules per ovary of 42 floral buds collected from each hybrid flower color morph, preserved in alcohol (70%), with the aid of a stereo microscope (VELAB VE-SI, USA). We used generalized linear mixed models (GLMM) using a Gaussian error distribution and logarithmic link function to assess morph differences (fixed effect) in flower size and number of ovules per flower, individual plant as a random effect, and measures as continuous response variables. All statistical analyses (here and below) were performed in R Studio version 3.2.2^77^.

### Nectar standing crops

Nectar standing crops and accumulated nectar throughout flower lifespans were quantified to determine reward availability for pollinators. Because pollinators probably respond to nectar standing crop, we extracted the nectar available in flowers that had been exposed to floral visitors and measured its volume and concentration. Data were collected in September and October 2016 from 20 individual plants (*N* = 153 flowers/hybrid flower color morph) at 3-h intervals, 10:00 (*N* = 51 flowers, 1–2 flowers sampled per plant/time interval), 13:00 (*N* = 51 flowers) and 16:00 (*N* = 51 flowers), to evaluate variation in the availability of nectar during the period of floral visitors activity. Nectar volume per flower was removed and measured by using calibrated micropipettes (5 µL) and a digital calliper (error: 0.1 mm). Sugar concentration (percentage sucrose) was measured by a hand-held pocket refractometer (range concentration 0–32° BRIX units; Atago, Tokyo, Japan), and the amount of sugar produced was expressed as milligrams of sugar^76^. A GLMM (Family = Gaussian, Link = logit) was also used to compare the effects of color morph and time of day (fixed effect) and plant identity (random effect) on nectar standing crops with nectar volume and amount of sugar as continuous response variables.

In a different group of 20 plants, buds about to open were randomly selected and bagged as explained above (*N* = 62 flowers/hybrid flower color morph) and excluded from floral visitors to let nectar accumulate. The accumulated nectar throughout the lifespan of individual flowers (6–13 d) was extracted one day before senescence. Nectar volume and sugar concentration were measured as explained above. A GLMM (Family = Gaussian, Link = logit) was used to compare the effects of color morph (fixed effect) and plant identity (random effect) on the amount of accumulated nectar (nectar volume and amount of sugar) as continuous response variables.

### Floral visitors

Upon flowering (from July to November 2017), we recorded the identity and foraging patterns of the floral visitors of each hybrid flower color morph. For these observations, we haphazardly selected 17 patches that contained 72 individual flowering plants (36 of each color morph), resulting in 192 hours of observation (20-min/plant, 1–5 h/day) at different days and times of day, from 10:00 until 16:00 h. We used binoculars (10 × 42, Celestron) and recorded the beginning of the observation as time zero and subsequent foraging events of each bumblebee and hummingbird visit as minutes from start time. We estimated three metrics of pollinator visitation per focal plant and hybrid flower color morph: elapsed time between the start of the observation period and when the first visit occurs, time spent per plant of each foraging bout, and the number of flowers visited per foraging bout. A floral visitation event was defined as the arrival of any visitor at one or more of the flowers of the target patch in which the visitor contacted the sexual organs of flowers. Total visits recorded from *Bombus* bumblebees were grouped and coded as visits from bumblebees, while visits from different hummingbird species were grouped under the hummingbird category. We used survival analysis^78^ to analyze the probability of each hybrid flower color morph being visited by bumblebees and hummingbirds. The non-parametric survival analysis tool was used because the actual time of all possible events to occur in a given focal plant is not always known during the focal observation. The Kaplan–Meier method of survival analysis was used to generate and adjust survival curves using the elapsed time variable that differed between pollinator types. The censored observations are plants that a pollinator had not yet visited 20-min after the start of the observation. The log-rank (Mantel–Cox) test was used for differences between pollinator survival curves. Visitation frequency and number of flowers visited per foraging bout (continuous response variables) by pollinator type (fixed effects) were analyzed using a GLMM (Family = Poisson, Link = logit). Plant identity was included as a random effect in the model. Differences between pollinator groups per time period were evaluated with Tukey multiple comparisons. Lastly, the foraging behavior variables were analyzed as a function of hybrid flower color morph and amount and availability of nectar through a linear regression model.

### Flower color measurement

The spectral reflectance of flowers of each putative parental species and hybrid flower color morph (20 flowers per species and color morph, *N* = 80 flowers) was measured to assess the consequences of color reflectance differences for pollinator attraction and plant reproduction. Flower reflectance of the apical, center and basal parts of the corolla were measured using a JAZ-EL 200 spectrometer with a UV-VIS deuterium tungsten halogen source (Ocean Optics Inc., Dunedin, FL, USA) connected to a computer running CLR version 1.1 (JAVA program). The light spectrum analyzed ranged from 300 to 700 nm, divided into 0.22-nm intervals, and the spectrometer sensor was fixed at an angle of 45° from the measuring areas between 215 and 1700 nm. We took all reflectance measurements at the same direction relative to the flower structure, using barium sulphate as the white standard, and analyzed spectral reflectance in four distinct wavebands (UV: 301–400 nm; blue: 400–510 nm; green: 510–605 nm; and red: 605–700 nm) for each part of the flowers. Petals were mounted on an adhesive tape to obtain a flat surface, thus minimizing reflectance variability (measured as percentage) due to uneven distances between the petals and the sensor.

We compared the flower colors from the tri- and tetrachromatic color vision systems of bumblebees and hummingbirds using the logarithm version of the receptor noise-limited model as in Bergamo *et al*.^21^. We used the standard photoreceptor sensitivities of bumblebees and hummingbirds adopting the oil droplet parameters from Hart & Vorobyev^79^, with noise values of 0.74, 0.67 and 0.61 as inputs in the bumblebee model for UV, blue and green photoreceptor types, respectively, while a violet sensitive (VS) vision system and noise values of 0.1, 0.07, 0.07 and 0.05 for the SWS1, SWS2, MWS and LWS photoreceptor type, respectively, for hummingbirds^66,80^. We also calculated the color loci of the flower colors in the respective color space models: the color hexagon for bees^81^ and the color tetrahedron for hummingbirds^65^, and the spectral purity in the color tetrahedron for hummingbirds only, since these are the two main color properties determining pollinator foraging decisions^21^.

Using PAVO^82^, we first generated a three-dimensional plot indicating the location of each point in the color tetrahedron, and then calculated the Cartesian coordinates (X, Y, Z) for the points in the tetrahedral color space, the angles theta and phi (h.theta, h.phi) in radians, which determine the hue of the color, the r vector (r.vec), which measures saturation or the distance from the achromatic center, the maximum r vector (r.max) achievable for the color’s hue, and the r.achieved, which measures the relative r distance from the achromatic center in relation to the maximum distance achievable (r.vec/r.max). In the lme4 package, we compared the pollinator’s chromatic discrimination in the average of the three floral parts between putative parental species and hybrid flower color morphs (fixed effect) performing a GLM (Family = Poisson, Link = logit). Additionally, we compared the pollinator’s chromatic discrimination between color morphs performing a one-sample *t*-test against the minimum discrimination threshold, and to evaluate differences in the chromatic detectability for each visual system.

### Pollinator effectiveness

We conducted two pollination experiments to test for differences in female reproductive success across the two hybrid flower color morphs and across pollinator types. For the open-pollination experiment, unbagged flowers of each color morph (*N* = 40 plants) were exposed to natural floral visitation until fruit initiation and quantified number of fruits produced per plant. Mature fruits from each flower color morph (*N* = 37 fruits per morph) were marked and collected two months later, and measured (length and width; digital vernier TRUPER) and weighed with an analytic balance (to the nearest 0.01 mg; VELAB VE-1000, linearity ± 0.02), and their seeds counted.

For the second pollination experiment, we compared the effectiveness of bumblebees and hummingbirds as pollinators of each hybrid flower color morph. Buds ready to open from 30 plants were chosen (*N* = 62 floral buds from each color morph) and once they opened, flowers were emasculated and bagged with bridal netting until stigmatic receptivity. One or the other pollinator type pollinated half of the plants of each color morph. Individuals (*N* = 4) of the most common visitor bumblebee species (*Bombus ephippiatus*) were captured, enclosed in test tubes and then used as pollen vectors. Pollination was accomplished by placing the test-tube entrance in front of the corolla of a donor flower with dehisced anthers and allowed the bumblebee to enter and leave the flower in a single event, and then repeated this procedure into the corolla of a receptive recipient flower. We used flowers as pollen donors only from one plant to minimize possible genetic factors and to simplify our experimental design. For hummingbirds, individuals of the most frequent floral visitor (*Selasphorus platycercus*; *N* = 3) were caught and used as pollen vector by allowing to insert its bill once into the corolla of a donor flower with dehisced anthers and then into the corolla of a receptive recipient flower, as in Salas-Arcos *et al*.^47^. After pollinations were performed the bumblebees and hummingbirds were released, and flowers remained bagged until fruit production, then fruits were weighed and the seeds per fruit produced were counted and measured by pollination treatment as explained above. True effectiveness of a pollinator is determined by the proportion of pollen grains picked up from one flower and deposited in the next^83^, but having flowers until hummingbird visited, and marking the visited flower, was not feasible. Pollen loads carried by the hummingbirds in hand are likely different from those carried by free hummingbirds because pollen from a specific flower (donor) may persist on hummingbirds for many flowers, mixing with pollen grains. Instead, hummingbirds only performed one pollination visit with pollen from one known flower to control for pollen donor and the amount of pollen carried and to simplify our experimental design. Only one plant was used as pollen donor to minimize possible confounding factors due to individual plant. As much as possible, we removed the residual pollen with a paintbrush prior to performing the next pollination. Nonetheless, decreased seed production from hand-pollinations may not be a rare event in experimental pollination experiments^84,85^. Pollen grains deposited on stigmas by hand pollination using hummingbirds as tools were not counted, but pollen loads were enough to fertilize most ovules^47,48^. Although pollen deposition is likely different between the open-pollination experiment and the hand pollination using hummingbirds as a tool, the number of seeds produced by pollinations with hummingbirds in hand in both morphs was greater than that obtained in flowers with open pollination (see Results). With this, the reliability of our manual pollinations is strengthened.

Permission to conduct the described field study was granted by the Mexican government (Secretaría del Medio Ambiente y Recursos Naturales, Dirección General de Vida Silvestre, SGPA/DGVS/02439/16). This collecting permit specifically allowed for the capture of birds. Manipulation of birds in the field was minimal. Birds were captured with mist nets and used as pollen vectors before they were released. All procedures with birds were carried out in accordance with the Guidelines for the Use of Wild Birds in Research proposed by the North American Ornithological Council and the ethics of experimental procedures were revised and authorized by the Animal Care and Use Committee under the Graduate Studies Committee (Posgrado en Ciencias Biológicas) of the Universidad Autónoma de Tlaxcala (UAT). While the field study involves a non-threatened or protected species, no specific permits are required for bird monitoring or observational studies as the one described here.

A GLMM model (Family = Poisson, Link = logit) was used to compare fruit production (discrete response variable) between hybrid flower color morphs (fixed effect) under natural conditions. The same GLMM model was used to assess differences between morphs in fruit length, fruit width, fruit weight and number of seeds per fruit, with color morph treated as a fixed effect and fruit traits and number of seeds produced per fruit treated as continuous response variables. Plant identity was included as a random effect in the model. To evaluate differences between bumblebees and hummingbirds in their effectiveness as pollinators, two GLMM models (with Gaussian error distribution) were performed with color morph, pollinator type and their interactions treated as fixed effects, and fruit length, fruit width, fruit weight, number of fruits or number of seeds produced per fruit treated as continuous response variables. Again, plant identity was included as a random effect in the model.

### Reproductive isolation

**Figure 5:**
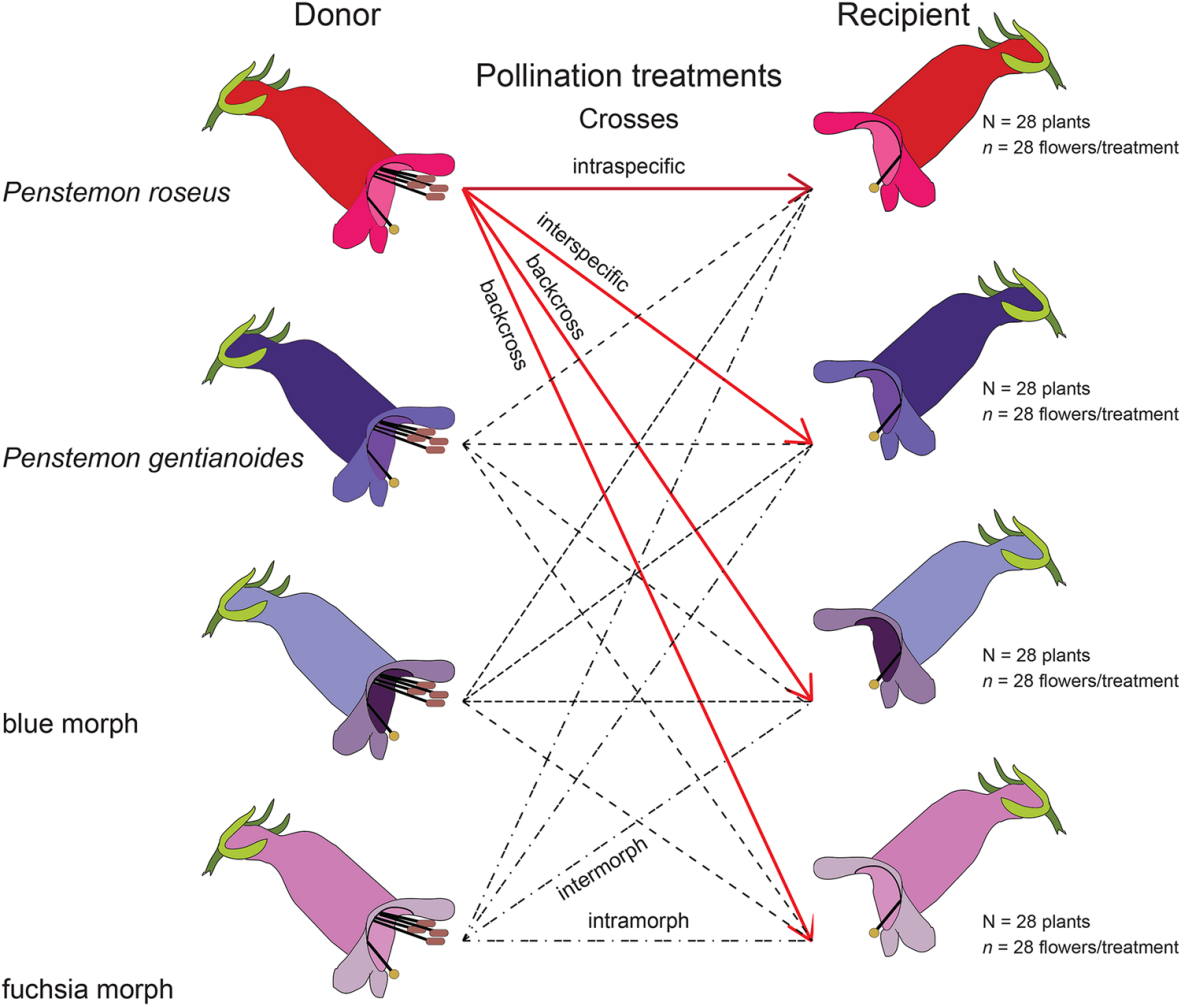
Experimental design of a hand-pollination experiment. The pollination treatments included intraspecific crosses, interspecific crosses, intermorph or intramorph crosses, and backcrosses among *Penstemon gentianoides*, *P. roseus*, and the ‘blue’ and ‘fuchsia’ hybrid flower color morphs. J. F. Ornelas drew the flower image in figure. The figure was drawn and edited in Adobe Illustrator CS6 v16.0.0 (Adobe Systems, Inc.).

A third hand-pollination experiment was conducted on *P. gentianoides*, *P. roseus*, and the hybrid flower color morphs to determine the existence of pre-zygotic post pollination barriers (post-pollination reproductive isolation). Crosses between *P. gentianoides* and *P. roseus*, and backcrosses in both directions from naturally occurring hybrid flower color morphs were achieved by rubbing one of the dehiscent anthers on the stigmas. The pollination treatments included: (1) intraspecific crosses (e.g., pollen of *P. roseus* on *P. roseus* stigmas), (2) interspecific crosses (e.g., pollen of *P. roseus* on *P. gentianoides* stigmas), (3) intermorph or intramorph crosses (between and within hybrid flower color morphs), and (4) backcrosses (between species and hybrid flower color morphs). We performed all possible crosses (16 pollination treatments, *N* = 28 flowers per treatment, one flower per plant; Fig. 5) in flowers emasculated prior to pollination and previously bagged to exclude pollinators, and re-bagged after each treatment. Due to its altitudinal range and abundance, interspecific pollinations that involved *P. roseus* as pollen receptor were conducted at lower elevation (2,900 m) and the rest near to the hybrid zone (3,800 m). For intraspecific pollinations, we used pollen from plants in different patches (at least 200 m apart) to avoid self-pollen or pollen from clonal individuals. Fruits produced from these crosses were quantified and collected and measured two months later. A GLM model (Family = binomial, Link = logit) was used to evaluate the probability of fruit formation (%) in manual crosses between hybrid flower color morphs and putative parental species. The full GLM model included donor and recipient plant, and their interactions treated as fixed effects and fruit production as a binary response variable. In addition, we compare color morphs and parental species^46–48^ in morphology, nectar, pollinator, and fruit production data. A GLM model (Family = Gaussian, Link = logit) was used to assess differences in fruit length, fruit width, fruit weight, and seed production between color morphs. A Tukey’s post hoc test was used for multiple comparisons among pairs of means of pollination treatments.

We quantified reproductive isolation (*RI*) to assess the contributions of post-zygotic barriers between parental species and hybrid flower color morphs (see Sobel and Chen^86^ for detailed explanations of *RI* calculations). We then compared *RI* values between hybrid flower color morphs, *P. gentianoides* and *P. roseus* following Sobel and Chen^86^: *R1*_4A_ = 1–2 * (*H*/*H* + *C*), in which *H* and *C* are fruit set of heterospecific (i.e., interspecific or intermoph) and conspecific (intraspecific or intramorph) crosses. In addition, we assessed the significance of pre-zygotic ecological isolating factors (pollinator assemblage) between morphs and species as in Sobel and Chen^86^: *RI*_4C_ = 1 – (*S*/*S* + *U*), where *S* refers to the extent of shared pollinators (i.e., lists of shared and unshared species) and *U* refers to the extent of unshared pollinators. The absolute and relative cumulative strength of each barrier was also quantified (calculations also provided in Sobel and Chen^86^) to determine which barrier is currently contributing the most to maintaining these hybrid flower color morphs and species in sympatry.

## Supplementary information

Supplementary information.

## Data Availability

The following information was supplied regarding data availability: Vouchers of each species and hybrid flower color morphs were deposited at the herbarium of the Centro de Investigación en Ciencias Biológicas, Universidad Autónoma de Tlaxcala, Mexico.
