# Supplementary material for: Pollinator divergence and pollination isolation between hybrids with different floral color and morphology in two sympatric Penstemon species
Source: Sci Rep. 2020 May 15;10:8126. doi: 10.1038/s41598-020-64964-8 (PMC7229217; doi:10.1038/s41598-020-64964-8)
Supplement: Supplementary file 1 — Supplementary information. [file 41598_2020_64964_MOESM1_ESM.pdf]

Pollinator divergence and pollination isolation between hybrids with different floral color and morphology in two sympatric *Penstemon* species

Scientific Reports

Juliana Cardona-Londoño, Carlos Lara & Juan Francisco Ornelas

✉ Juan Francisco Ornelas, Departamento de Biología Evolutiva, Instituto de Ecología, A.C. (INECOL), Carretera antigua a Coatepec No. 351, El Haya, Xalapa, Veracruz 91070, Mexico; francisco.ornelas@inecol.mx

Supplementary Fig. S1. Nectar standing crops and accumulated nectar in *Penstemon* 'blue' and 'fuchsia' flowers throughout the day. Nectar standing crops for (A) volume (μL per flower) and (B) amount of sugar (mg of sugar/ml per flower) in *Penstemon* 'blue' and 'fuchsia' flowers throughout the day. (C) Accumulated nectar volume and (D) amount of sugar throughout the flower lifespan. Data (means ± SE) with the same superscript letters are not significantly different among groups.

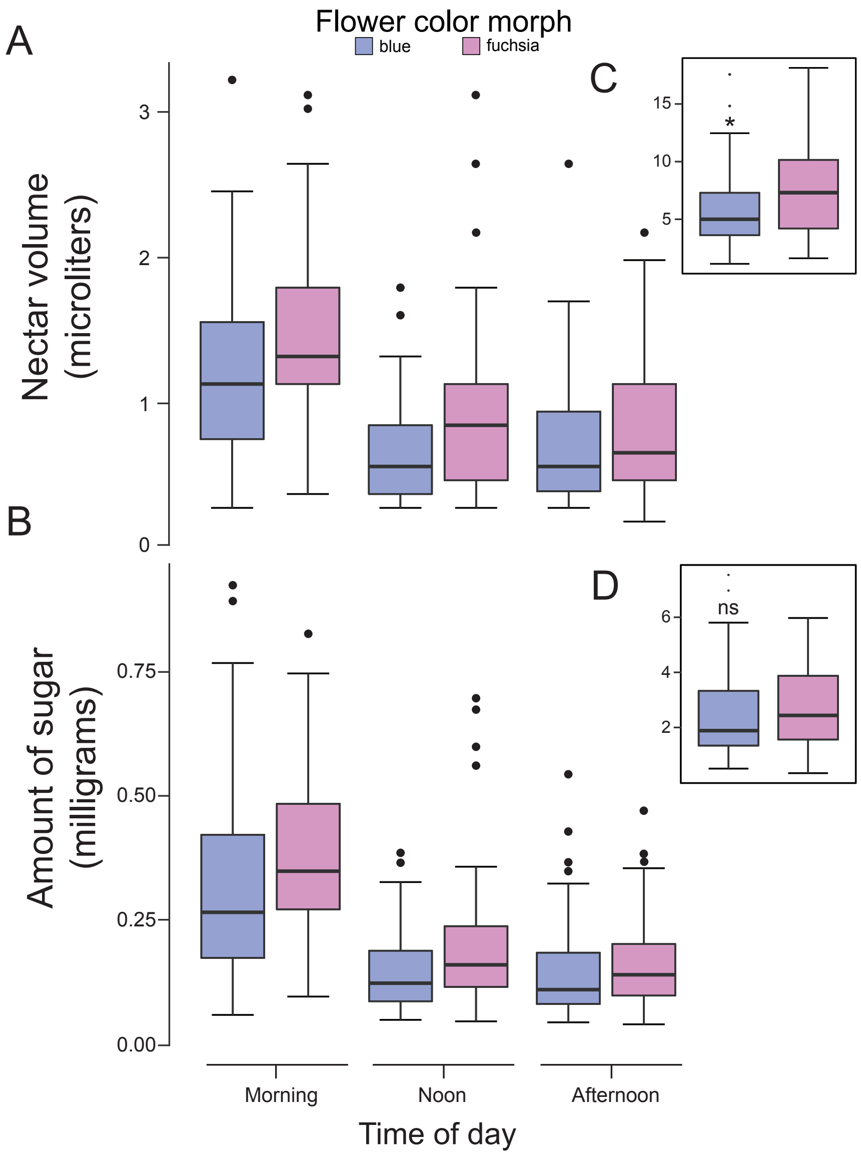

Supplementary Fig. S2. Fruit traits and seed number for *Penstemon* blue and fuchsia flowers naturally (open pollination) (A) or experimentally pollinated by bumblebees (*Bombus ephippiatus*) or hummingbirds (*Selasphorus platycercus*) (B).

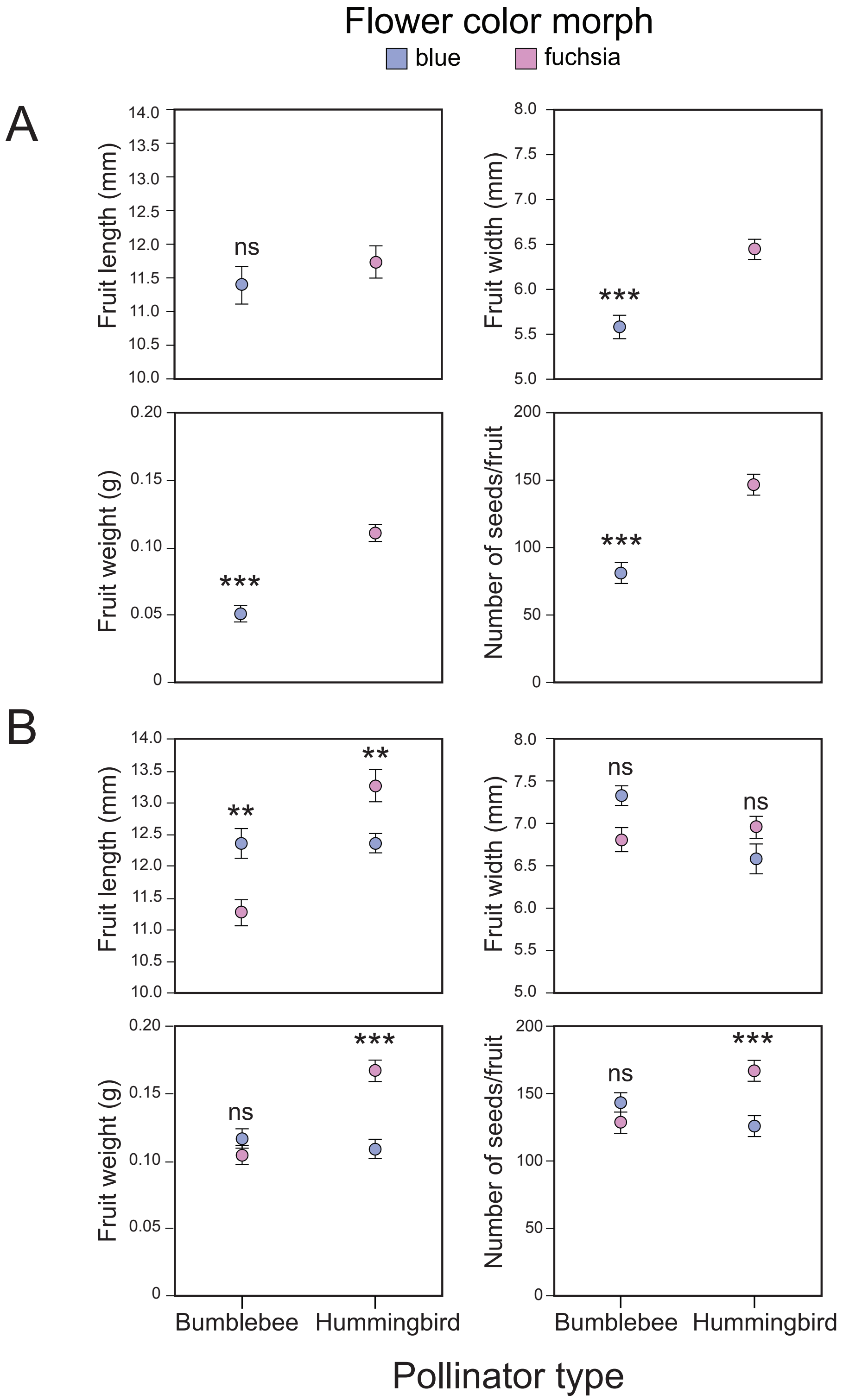

Supplementary Fig. S3. Fruit traits (fruit length, fruit width, fruit weight) and seed number in conspecific backcrosses or heterospecific crosses for blue, fuchsia, *Penstemon gentianoides*, and *P. roseus* recipient flowers that set fruit.

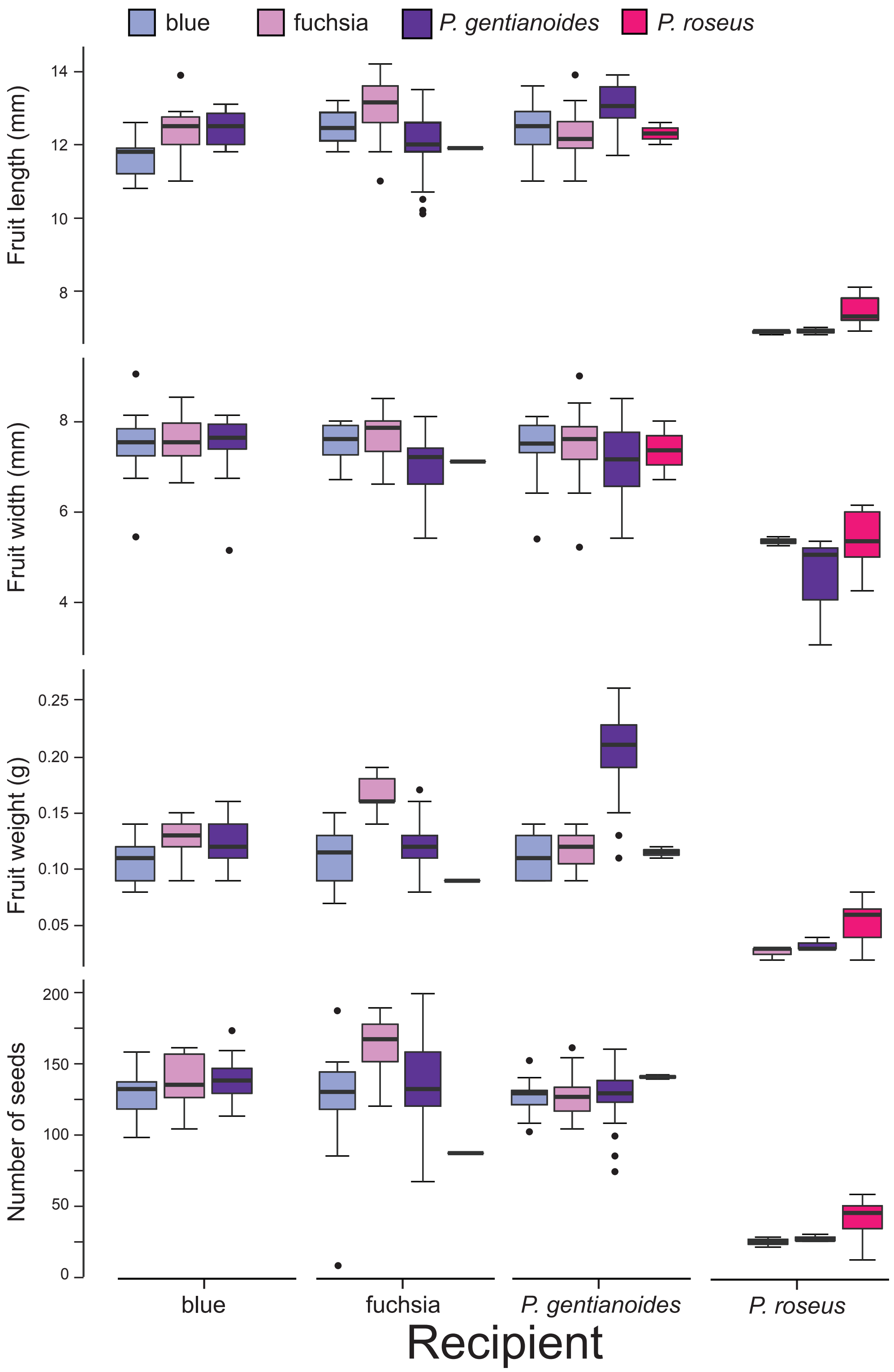

Supplementary Fig. S4. Contrasts between putative parental species, *Penstemon gentianoides* and *P. roseus*, and hybrid flower color morphs in flower (corolla length, corolla-entrance width, stamen length) and nectar traits (volume of nectar, amount of sugar).

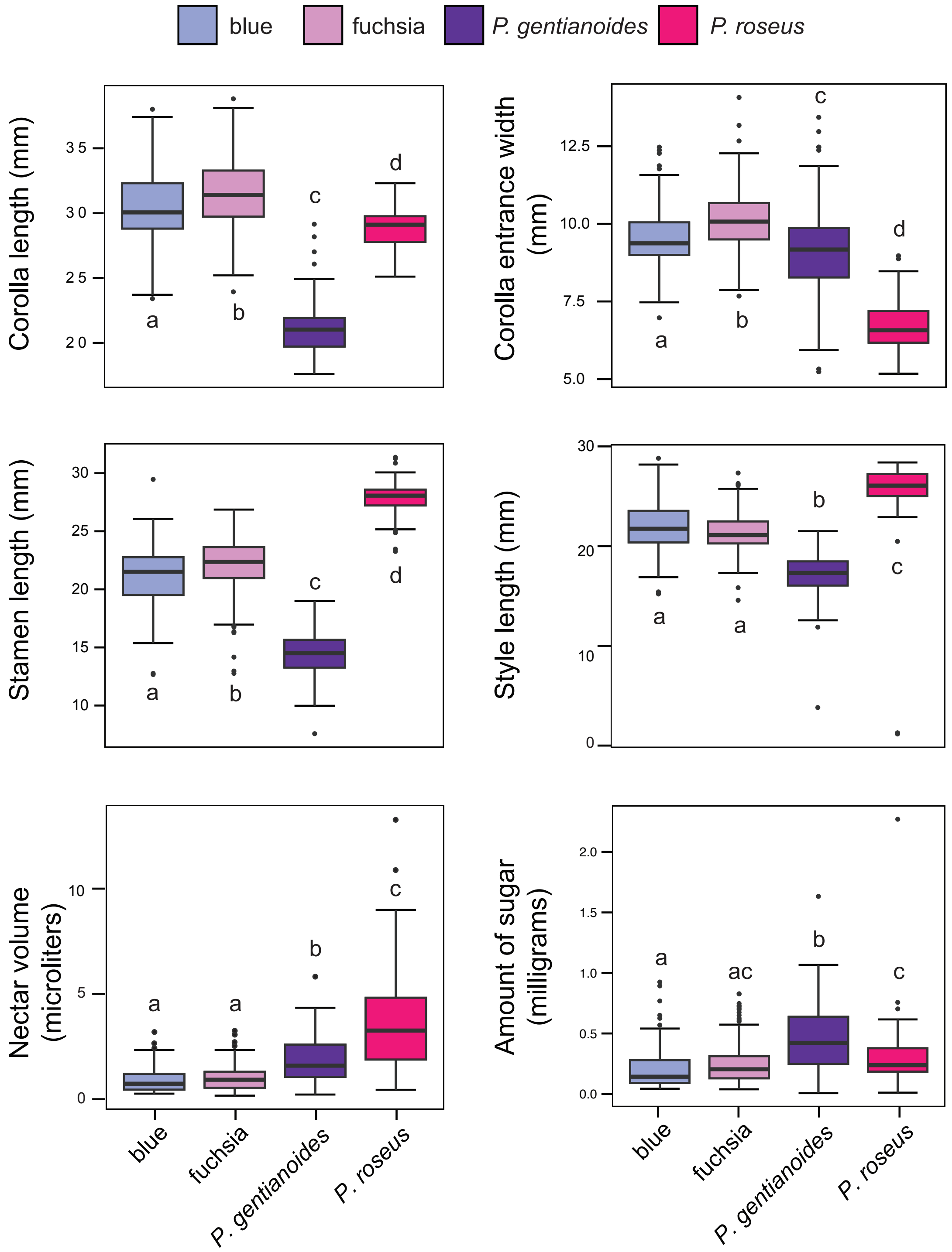

Table S1. Flower measurements (mm) of naturally growing individuals of *Penstemon* hybrid flower color morphs in a montane temperate forest at La Malinche National Park (LMNP), Tlaxcala, Mexico.

**Variable**	***N***	**fuchsia**	***N***	**blue**	**GLMM (color morph)**
Corolla tube length	134	31.73 ± 0.24 (23.5–38.1)	134	30.45 ± 0.25 (24.03–38.9)	$F = 18.22$***
Corolla-entrance width	134	10.13 ± 0.08 (7.0–12.5)	134	9.56 ± 0.08 (7.7–14.1)	$F = 26.92$***
Corolla-entrance height	134	27.32 ± 0.23 (20.0–36.7)	134	25.98 ± 0.25 (20.4–35.1)	$F = 16.25$**
Filament (short) length	134	18.81 ± 0.16 (12.3–25.1)	134	19.34 ± 0.20 (12.7–24.4)	$F = 4.74$*
Filament (long) length	134	21.67 ± 0.17 (15.9–28.8)	134	22.33 ± 0.20 (15.3–27.4)	$F = 7.28$**
Style length	134	21.92 ± 0.22 (12.7–29.5)	134	21.20 ± 0.21 (12.8–26.9)	$F = 5.53$*

Data are means ± SE and ranges in parentheses. Relationships were modelled using a generalized linear model (GLMM), with hybrid flower color morph treated as fixed effect, plant as random effect and measures as continuous response variables. *** $P < 0.0001$, ** $P < 0.001$, * $P < 0.05$.

Table S2. Nectar standing crops (morning, noon, afternoon) and accumulated nectar in *Penstemon* fuchsia and blue morph flowers.

**Variable**	***N***	**fuchsia**	***N***	**blue**
Morning	51		51	
Volume (μL)		1.49 ± 0.09		1.19 ± 0.08
		(0.37–3.11)		(0.28–3.21)
Amount of sugar (mg)		0.38 ± 0.02		0.32 ± 0.02
		(0.09–0.82)		(0.05–0.92)
Noon	51		51	
Volume (μL)		0.92 ± 0.08		0.70 ± 0.05
		(0.28–3.11)		(0.28–1.79)
Amount of sugar (mg)		0.20 ± 0.02		0.14 ± 0.01
		(0.04–0.69)		(0.04–0.38)
Afternoon	51		51	
Volume (μL)		0.79 ± 0.06		0.72 ± 0.06
		(0.18–2.17)		(0.28–2.64)
Amount of sugar (mg)		0.16 ± 0.01		0.14 ± 0.01
		(0.03–0.47)		(0.04–0.54)
Accumulated	62		62	
Volume (μL)		7.63 ± 0.49		5.98 ± 0.48
		(1.61–18.13)		(1.13–17.56)
Amount of sugar (mg)		2.80 ± 0.20		2.43 ± 0.22
		(0.33–5.97)		(0.49–7.54)

Data are means ± SE and ranges in parentheses. Volume expressed in microliters (μL) per flower and amount of sugar as mg of sugar/mL per flower. Accumulated nectar corresponds to the volume and amount of sugar per flower throughout their lifespan.

Table S3. Receiver-centric variables (hue and chroma) of a tri- and tetrachromatic color vision systems for bee and bird vision, respectively, using a sensory-based analysis.

	**Relative cone stimulation (%)**				**Tetrahedral color space**			**Hue (h, radians)**		**Chroma (r)**	
	U	S	M	L	X	Y	Z	h.theta	h.phi	r	rA
Hummingbird											
fuchsia	0.1542	0.1631	0.2918	0.3908	0.1394	0.0105	–0.0958	0.0893	–0.6084	0.1699	0.3854
blue	0.2059	0.2049	0.2706	0.3186	0.0697	0.0063	–0.0441	0.1074	–0.5630	0.0831	0.1934
P. roseus	0.0658	0.1052	0.3132	0.5158	0.2514	0.0019	–0.1842	0.0174	–0.6395	0.3121	0.7369
P. gentianoides	0.1572	0.1675	0.2900	0.3854	0.1334	0.0096	–0.0928	0.0860	–0.6084	0.1631	0.3742
Bumblebee											
fuchsia	N/A	0.2486	0.2921	0.4594	0.1183	–0.1038	N/A	–0.7292	N/A	0.1581	N/A
blue	N/A	0.2764	0.3315	0.3922	0.0429	–0.0698	N/A	–1.0228	N/A	0.0822	N/A
P. roseus	N/A	0.1295	0.2078	0.6628	0.3217	–0.2497	N/A	–0.6843	N/A	0.4081	N/A
P. gentianoides	N/A	0.2358	0.2937	0.4705	0.1250	–0.1195	N/A	–0.8242	N/A	0.1745	N/A

U = UV receptor; Visual stimulation from short (S), medium (M) and long (L) waves. Cartesian coordinates (X, Y, Z) for the points in the tetrahedral color space; hue of the color: theta and phi (h.theta, h.phi) angles in radians; r vector (r.vec) of the chroma = saturation or the distance from the achromatic center, r.max = maximum r vector achievable for the color's hue; r.achieved (rA) = relative r distance from the achromatic center in relation to the maximum distance achievable (r.vec/r.max).

Table S4. Contribution of assessed pre-pollination and post-pollination barriers to reproductive isolation (*RI*) between hybrid flower color morphs (blue and fuchsia), *P. gentianoides* (PG) and *P. roseus* (PR) for the studied reproductive barriers.

**Isolating barrier**		**Raw values**		**Sobel and Chen *RI* value**	**Absolute cumulative strength**	**Relative cumulative strength**	***RI* total**
(a) blue vs. fuchsia							
Fruit set		Heterospecific	Conspecific				
	blue	0.46	0.54	0.08	0.073	0.204	0.3589
	fuchsia	0.48	0.52	0.04	0.035	0.098	
Pollinator isolation		Shared	Unshared				
		0.75	0.25	0.25	0.25	0.696	
(b) blue vs. PR							
Fruit set		Heterospecific	Conspecific				
	blue	0	1	1	0.04	0.4	1
	PR	0	1	1	0	0	
Pollinator isolation		Shared	Unshared				
		0.40	0.60	0.60	0.60	0.60	
(c) blue vs. PG							
Fruit set		Heterospecific	Conspecific				
	blue	0.45	0.55	0.10	0.06	0.087	0.6929
	PG	0.47	0.53	0.06	0.032	0.047	
Pollinator isolation		Shared	Unshared				
		0.44	0.56	0.60	0.60	0.865	
(d) fuchsia vs. PR							
Fruit set		Heterospecific	Conspecific				
	fuchsia	0.12	0.88	0.76	0.489	0.492	0.9951
	PR	0.04	0.96	0.92	0.105	0.106	
Pollinator isolation		Shared	Unshared				
		0.60	0.40	0.40	0.40	0.402	
(e) fuchsia vs. PG							
Fruit set		Heterospecific	Conspecific				
	blue	0.42	0.58	0.16	0.111	0.18	0.6168
	fuchsia	0.54	0.55	0.009	0.005	0.009	
Pollinator isolation		Shared	Unshared				

		0.50	0.50	0.50	0.50	0.81	
(f) PR vs. PG							
Fruit set		Heterospecific	Conspecific				
	PR	0.08	0.92	0.84	0.269	0.027	0.9966
	PG	0.01	0.90	0.80	0.026	0.026	
Pollinator isolation		Shared	Unshared				
		0.30	0.70	0.70	0.70	0.702	

Isolation components generally vary from zero (no barrier) to one (complete isolation). Contributions to total reproductive isolation were calculated for sequential reproductive barriers, with the sum of contributions equaling total isolation. Sobel and Chen's[86] methodology was used for *RI*; see Sobel and Chen's[86] Supporting Information for detailed explanations of *RI* calculations.
